# Transcriptomic analysis of thermotolerant yeast *Kluyveromyces marxianus* in multiple inhibitors tolerance†

**DOI:** 10.1039/c8ra00335a

**Published:** 2018-04-17

**Authors:** Dongmei Wang, Dan Wu, Xiaoxue Yang, Jiong Hong

**Affiliations:** School of Life Sciences, University of Science and Technology of China Hefei Anhui 230027 P. R. China hjiong@ustc.edu.cn +86 551 63601443 +86 551 63600705

## Abstract

During pretreatment of lignocellulosic biomass, toxic compounds were released and inhibited the growth and fermentation of microorganisms. Here the global transcriptional response of *K. marxianus* to multiple inhibitors including acetic acid, phenols, furfural and HMF, at 42 °C, was studied, *via* RNA-seq technology. Genes involved in the glycolysis pathway, fatty acid metabolism, ergosterol metabolism and vitamin B6 and B1 metabolic process were enriched in the down-regulated gene set, while genes involved in TCA cycle, respiratory chain, ROS detoxification and transporter coding genes were enriched in the up-regulated gene set in response to the multiple inhibitors stress. Further real time-PCR results with three single inhibitor stress conditions showed that different transporters responded quite differently to different inhibitor stress. Coenzyme assay results showed that the level of NAD^+^ was increased and both NADH/NAD^+^ and NADPH/NADP^+^ ratio decreased. Furthermore, genes involved with transcription factors related to carbohydrate metabolism, sulfur amino acids metabolism, lipid metabolism or those directly involved in the transcriptional process were significantly regulated. Though belonging to different GO categories or KEGG pathway, many differentially expressed genes were enriched in maintaining the redox balance, NAD(P)^+^/NAD(P)H homeostasis or NAD^+^ synthesis, energy production, and iron transportation or metabolism. These results suggest that engineering these aspects represents a possible strategy to develop more robust strains for industrial fermentation from cellulosic biomass.

## Abstract 2

Global transcriptional response of *K. marxianus* to multiple inhibitors including acetic acid, phenols, furfural and HMF at 42 °C.

## Graphical abstract

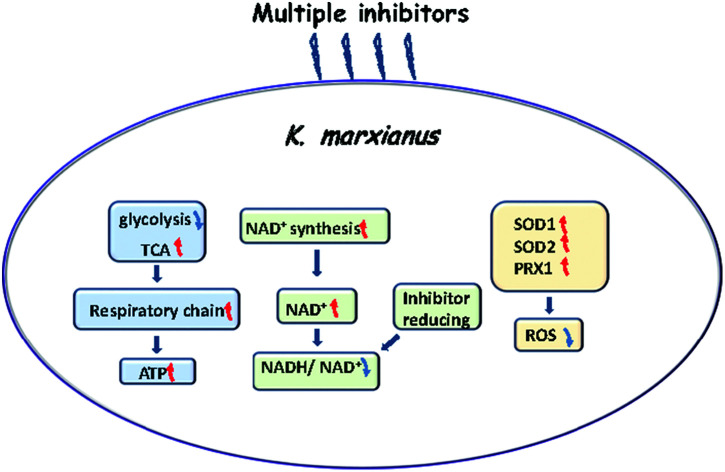

## Introduction

Lignocellulosic biomass has the potential to contribute substantially to future global energy demands, because of its low cost, large-scale availability, not competing with food production, and high potential to reduce greenhouse gas emission.^1–4^ Pretreatment is essential for releasing fermentable sugars from lignocellulose biomass. However, during harsh pre-treatment processes, various small molecules such as weak acids, furan aldehydes, and phenolic compounds (referred to as “fermentation inhibitors”), are generated from partial over-degradation of lignocellulose and inhibit consequent cell growth and microbial fermentations.^5^ The inhibitor tolerance is one of the important parameter for effective fermentation process. While prior studies are mostly focused on characterization of genetic mechanisms for yeast stress response to individual inhibitory compounds, cellulosic hydrolysates contain multiple fermentation inhibitors with distinct toxicity mechanisms rather than a single inhibitor. It was found that different mechanisms could be adopted by the yeast to resist hydrolysates inhibitors, *e.g.* acetic acid, furfural, and 5-hydroxymethylfurfural (HMF).^6^ However, there is still limited information on what genetic perturbation targets could be elicited to improve yeast resistance to mixed fermentation inhibitors. Therefore, a better understanding of genetic regulatory networks underlying the resistance to multiple lignocellulosic-derived fermentation inhibitors is needed to develop strains with enhanced tolerance to cellulosic hydrolysates. Considering the fact that various inhibitors often coexist in the hydrolysate and could cooperate with each other to become even more toxic to yeast than existing alone (*i.e.*, synergistic stress), the knowledge on how yeast cells reprogram their metabolism in response to lignocellulosic-derived fermentation inhibitors is of particular interests to biofuel and biochemical production.

*Kluyveromyces marxianus* is considered as a ‘generally regarded as safe’ (GRAS) microorganism. Though the genome of *K. marxianus* was much smaller (less than 5000 open reading frames)^7^ than that of *S. cerevisiae* (over 6000 genes),^8^ it has advantages such as short generation time and high growth rate at elevated temperatures (0.86–0.99 h^−1^ at 40 °C), with an upper growth limit of 52 °C of some strains.^9^*K. marxianus* also has the intrinsic fermentation capacity to utilize various substrates including xylose.^10–12^ Therefore, there are increasing applications of *K. marxianus* in high temperature fermentation with lignocellulosic hydrolysates. However, the knowledge of its stress physiology is scarce. Moreover, *K. marxianus* natively exhibited higher assimilation rates for aldehydes such as furfural, HMF, vanillin *etc.*, compared to glucose-fermenting microorganisms such as *Klebsiella pneumoniae*, *Saccharomyces cerevisiae*, and *Zymomonas mobilis* with no genetic modification.^13^ Our study also showed that *K. marxianus* could ferment with non-detoxified diluted acid pretreated corncob to produce ethanol and xylitol and possess considerate inhibitors tolerance especially to furfural and HMF.^14^ However, compared with vast information of various inhibitors tolerance in *S. cerevisiae*, there is very limited information on *K. marxianus* with the resistance mechanism to the fermentation inhibitors. Therefore, transcriptomic analysis of the tolerance response of lignocellulosic hydrolysates inhibitors or fermentation inhibitors will be much helpful in *K. marxianus* fermentation study.

Although genome sequences of several *K. marxianus* strains have been published,^15–17^ detailed reports on the transcriptional analysis of *K. marxianus* with various fermentation perturbations are still very limited. Lertwattanasakul *et al.* conducted transcriptome analyses of *K. marxianus* DMKU 3-1042 to identify genes related to growth with glucose at 45 °C and with xylose at 30 °C. Gao *et al.* reported the transcriptional analysis of *K. marxianus* for ethanol production from inulin.^18^ Up to now, no detailed transcriptional analysis of *K. marxianus* is available with lignocellulosic-derived fermentation inhibitors at elevated temperature (>30 °C). Comparing with the vast transcriptional analysis reports on *S. cerevisiae*, the study of *K. marxianus* is very limited which hindered the future development of *K. marxianus* application in industry.

Here we conducted transcriptomic analysis of *K. marxianus* at elevated temperature (42 °C) with or without three main lignocellulosic-derived fermentation inhibitors including acetic acid, furfural, HMF and phenols by next-generation sequencing technology for RNA (RNA-seq). The transcriptional comparison provides useful information on the molecular basis of genome-wide microbial responses to the mixed fermentation inhibitors, including the molecular basis of the central carbon metabolism, mitochondrial respiratory chain, redox homeostasis, MSN2/4 mediated stress response element (STRE)-controlled genes, fatty acid and ergosterol metabolism, alanine, aspartate and glutamate metabolism, vitamin B6 and B1 metabolism, together with various transporters genes which would facilitate the development of *K. marxianus* in the industrial application. Results of this study will aid dissection of lignocellulosic hydrolysate inhibitors tolerance mechanisms in yeast and metabolic engineering efforts for more tolerant strain development.

## Materials and methods

### Reagents and strains

All chemicals used were of analytical grade or higher. d-Glucose was obtained from Sangon Biotech Co. (Shanghai, China). The yeast extract and peptone were purchased from Oxoid (Oxoid Ltd., Basingstoke, Hampshire, England). *K. marxianus* YHJ010 was the *TRP1*, *LEU2* and *URA3* auxotrophic strain of NBRC1777 (ref. 19) and was cultivated in YPD medium (1% w/v yeast extract, 2% w/v bacto peptone, 2% w/v glucose) at 42 °C.

### Samples preparation and transcriptome analysis

#### Cell growth conditions

*K. marxianus* YHJ010 was pre-cultivated in 5 ml of YPD medium at 42 °C overnight. Then the cells were transferred into 500 ml Erlenmeyer flasks containing 100 ml of the YPD medium with or without mixed inhibitors containing 0.7 g l^−1^ furfural, 0.7 g l^−1^ HMF, 3.0 g l^−1^ acetate acid and 0.28 g l^−1^ phenols (4-hydroxybenzaldehyde, syringaldehyde, catechol and vanillin with 0.07 g l^−1^ each compound) with initial OD_600_ of 0.5 and cultivated at 42 °C with shaking at 250 rpm in an orbital shaker until OD_600_ of 6 (the mid-exponential phase of growth). In the case of individual inhibitor condition, acetic acid stress condition with 2.5 g l^−1^ acetic acid, furfural stress condition with 1.5 g l^−1^ furfural, and phenols stress condition with 0.8 g l^−1^ phenols (4-hydroxybenzaldehyde, syringaldehyde, catechol and vanillin with 0.2 g l^−1^ each compound), respectively. Yeast cells were then recovered when OD_600_ reached 6 and rapidly frozen in liquid nitrogen and kept at −70 °C until the subsequent RNA extraction step. Cell growth was monitored by determining the optical density (OD_600_) with a Hitachi UV-2550 Spectrophotometer.

#### RNA isolation, preparation of cDNA library and sequencing

Total RNA from yeast was extracted following the standard protocol of TRIZOL Reagent (Invitrogen, Carlsbad, CA, USA). mRNA was isolated from total RNA using Magnetic Oligo-dT beads, fragmented into short fragments and then used to synthesize first-strand cDNA with random primers. RNase H, buffer, dNTPs and DNA polymerase I (TaKaRa) was used to synthesize the second-strand cDNA. Sequencing adapters were ligated to short fragments and resolved by agarose gel electrophoresis. Suitable fragments were purified and subsequently amplified by PCR to create the cDNA library.

The cDNA was then shotgun sequenced (101-bp paired-end read) with the Illumina HiSeq 4000 instrument (Illumina, San Diego, CA, USA) using a customer sequencing service (Majorbio Co., Ltd, Shanghai, China).

#### Genome annotation and bioinformatics analysis

Adaptor sequences, empty reads, and low-quality sequences were removed from the raw reads, and the resulting clean reads were mapped to the reference genome of *Kluyveromyces marxianus* NBRC1777 from GenBank with accession no. AP014599-AP014607 (ref. 7) using TopHat (http://tophat.cbcb.umd.edu). The whole-genome sequences above were annotated according to the Gene Ontology (GO) database, Kyoto Encyclopedia of Genes and Genomes (KEGG) database.

For gene function annotation, obtained unigene sequences were annotated by searching in various protein databases, including the National Center for Biotechnology information (NCBI) nonredundant protein (Nr) database, the NCBI non-redundant nucleotide sequence (Nt) database, Cluster of Orthologous Groups of proteins (COG), Search Tool for the Retrieval of Interacting Genes (STRING), Gene Ontology (GO) and the Kyoto Encyclopedia of Genes and Genomes (KEGG). In addition, information for the differentially expressed genes (DEGs) was subjected to GO and KEGG significant enrichment analyses to identify biological functions and metabolic pathways in which these genes participate.

For differential gene expression analysis, reads per kilobase of exon model per million mapped reads (RPKM) was used as a value of normalized gene expression. Statistical comparison of RPKM values between the samples was conducted using a web tool Cuffdiff (http://cole-trapnell-lab.github.io/cufflinks/cuffdiff/index.html). Genes were considered differentially expressed in a given library when *p*-value < 0.05 and a greater than two-fold change in expression across libraries observed.

### Real-time PCR analysis

Total RNA was isolated using a yeast total RNA extraction kit (Sangon Biotech Co. Shanghai, China). Isolated RNA was treated with RNase-free DNase I (Toyobo, Osaka, Japan) at 37 °C for 15 min to remove the potentially contaminated genomic DNA. cDNA was synthesized by the ReverTra Ace qPCR RT Master Mix kit (Toyobo, Osaka, Japan). The reverse transcription reaction was performed in an Arktik thermal cycler (Thermo Fisher Scientific, Waltham, MA, USA) at 37 °C for 15 min, 50 °C for 5 min, and denaturing at 98 °C for 5 min. The synthesized cDNA was quantitatively determined by Nanodrop 2000 (Thermo Fisher Scientific, West Palm Beach, Florida, USA). Real-time PCR was conducted on Bio-Rad iCycler iQ (Bio-Rad, Hercules, CA, USA) with THUNDERBIRD SYBR qPCR mix kit (Toyobo, Osaka, Japan). Gene *ACT1* of *K. marxianus* NBRC1777 was used as an internal control. The primers for RT-PCR are shown in Table S4.† The cycle threshold values (*C*_T_) were determined and the relative fold differences were calculated by the 2^−ΔΔCT^ method^20^ using *ACT1* as the endogenous reference gene. Samples were run in triplicate in a 96-well plate, and each experiment was repeated three times. The value of qPCR presented is the mean of the triplicate results.

### Measurement of the intracellular coenzyme contents

Intracellular NAD(P)H and NAD(P)^+^ were extracted using EnzyChrom™ NAD(P)^+^/NAD(P)H assay kit (BioAssay Systems, Hayward, California, USA) following the manufacturer's instruction. A 10 ml sample of yeast culture was withdrawn and sprayed into quenching solution (60% methanol and 70 mM HEPES). Then, the quenched pellets were resuspended in acid extraction buffer or base extraction buffer (BioAssay Systems), after which they were incubated at 65 °C for 5 min in a water bath to extract oxidized pyridine nucleotides or reduced pyridine nucleotides, respectively. The relative amounts of NAD^+^, NADH, NADP^+^, and NADPH in each extract were then quantified by enzymatic methods using a NADP^+^-specific glucose dehydrogenase cycling reaction and a NAD^+^-specific lactate dehydrogenase cycling reaction, in which the formed NADPH or NADH reduces a formazan (MTT) reagent (BioAssay Systems).^21,22^ At OD_600_ = 1, the concentration of the cells was equivalent to 0.411 g l^−1^ dry cell weight (DCW).^23^

## Results

### Overview of transcriptional data with mixed fermentation inhibitors by RNA-seq

**Fig. 1:**
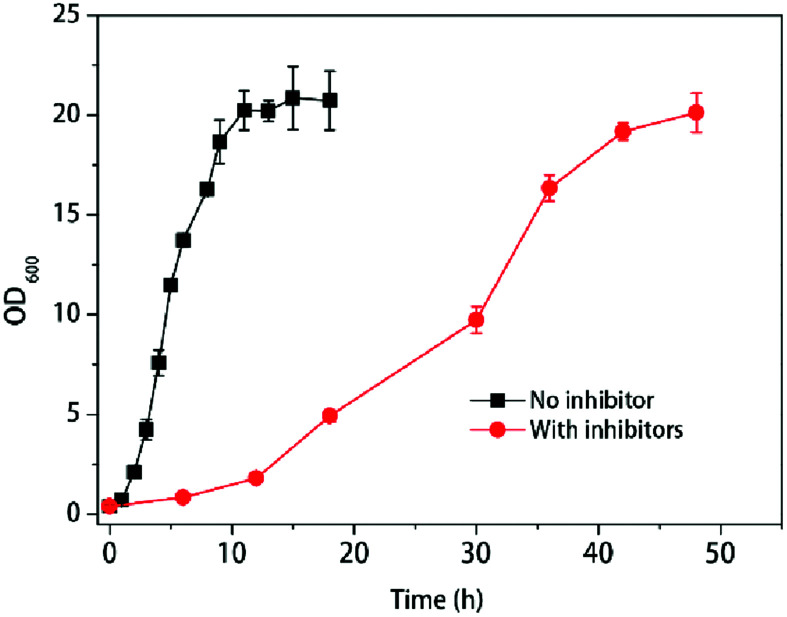
Response of cell growth to multiple inhibitors at 42 °C. Values shown are mean with SD (*n* = 3).

Yeasts reacted differently with various inhibitors in pretreated hydrolysate. To mimic the real fermentation procedure, here we used the mixed three main group inhibitors in the lignocellulosic pretreated hydrolysate (furfural, acetic acid, phenols) and the compound concentrations were used as the previous report^24–26^ with minor modification according to the growth of *K. marxianus* YHJ010 which derived from *K. marxianus* NBRC1777.^19^ With the treatment of multiple inhibitors, the cells growth was slower than those without inhibitors, as shown in Fig. 1.

The alteration of genome-wide gene expression was analyzed by RNA-seq analysis of *K. marxianus* YHJ010 with or without multiple inhibitors treatment. A total of 13 622 794 and 17 697 296 clean reads were obtained from the RNA of *K. marxianus* with or without inhibitors treatment, respectively, and over 91% were uniquely mapped to the reference genome (Table S1†).

### Identification of differentially expressed genes and validation by qPCR

The levels of gene expression, normalized as reads per kilobase of exon model per million mapped reads (RPKM), were applied to the fold changes of the differentially expressed genes (DEGs, with absolute fold changes (FC) ≥ 2; *p* ≤ 0.05). Under the stress with multiple inhibitors, 451 transcripts were identified to have different expression levels compared to those without stress. Among them, 279 genes were up-regulated and 172 genes were down-regulated (statistical data from the differentially expressed genes, data not shown). We then performed GO enrichment analysis on these DEGs. As shown in Table S2 and Fig. S1,† most DEGs in glycolytic process (GO:0006096), monocarboxylic acid metabolic process (GO:0032787), pyruvate metabolic process (GO:0006090) and NADP metabolic process (GO:0006739) *etc.* were down-regulated under the multiple inhibitors stress; on the other hand, most DEGs in tricarboxylic acid cycle (GO:0006099), transmembrane transport (GO:0055085), single-organism transport (GO:0044765), oxidative phosphorylation (GO:0006119), transporter activity (GO:0005215), respiratory chain (GO:0070469), substrate-specific transporter activity (GO:0022892), cellular response to chemical stimulus (GO:0070887) and response to stress (GO:0006950) *etc.* were up-regulated.

We also performed KEGG enrichment analysis on these DEGs. The ratio of DEGs/background genes indicates the effects of the DEGs in the specific KEGG pathway (background genes). As shown in Table S3 and Fig. S2,† the expression of most genes in degradation of aromatic compounds (Ko0120), retinol metabolism (Ko00830), drug metabolism (Ko00983), glycolysis/gluconeogenesis (Ko00010), methane metabolism (Ko00680), carbon fixation in photosynthetic organisms (Ko00710), citrate cycle (Ko00020) *etc.* were significantly regulated under the inhibitors stress, suggesting that the yeasts boot up the detoxification response to deal with the fermentation inhibitors in environment.

To confirm the reliability of data from RNA-seq, sixteen genes involved in various pathways were selected for a quantitative real-time PCR (qPCR) comparison of their expression levels. As illustrated in Table 1, although the relative expression levels (the fold changes, shown as the sign of the log_2_ transformed fold change (FC) values, log_2_ FC(I/C)) of each selected gene were different between RNA-seq and qPCR, the trends of up- or down-regulation of all genes chosen were the same, which consequently demonstrated the accuracy of the trends of gene expression change obtained by RNA-seq (Table 1).

**Table 1:** Comparison of log_2_ FC(I/C) in DEGs between RNA-seq and qPCR^a^

Gene ID	NR description	RNA-seq	qPCR
KMAR_10772	Uncharacterized abhydrolase domain-containing protein YGR015C	10.45	7.76
KMAR_80057	DNA-directed RNA polymerase II subunit RPB1	7.58	5.76
KMAR_50053	Uncharacterized protein IRC8	5.85	3.75
KMAR_10795	Ribosyldihydronicotinamide dehydrogenase [quinone]	5.73	5.26
KMAR_80139	Carbonic anhydrase	5.22	6.52
KMAR_40093	Siderophore iron transporter ARN2	5.19	7.71
KMAR_50521	Succinate dehydrogenase [ubiquinone]	4.89	5.37
KMAR_10054	Putative nitroreductase HBN1	4.6	4.29
KMAR_30337	ATP-dependent permease PDR12	4.56	5.39
KMAR_10790	Major facilitator superfamily	3.31	2.90
KMAR_20248	Putative sialic acid transporter	3.03	5.30
KMAR_80266	Myo-inositol transporter 2	2.66	3.24
KMAR_60075	Carboxylic acid transporter protein homolog	2.59	5.18
KMAR_50130	Multidrug resistance protein fnx1	2.05	2.72
KMAR_20313	Riboflavin transporter MCH5	−2.01	−1.86
KMAR_70277	Copper transport protein CTR1	−2.8	−2.06

a I and C represent samples of yeast grown on medium with or without multiple inhibitors in YPD under aerobic condition.

### Characterization of DEGs involved in central carbon metabolism

**Fig. 2:**
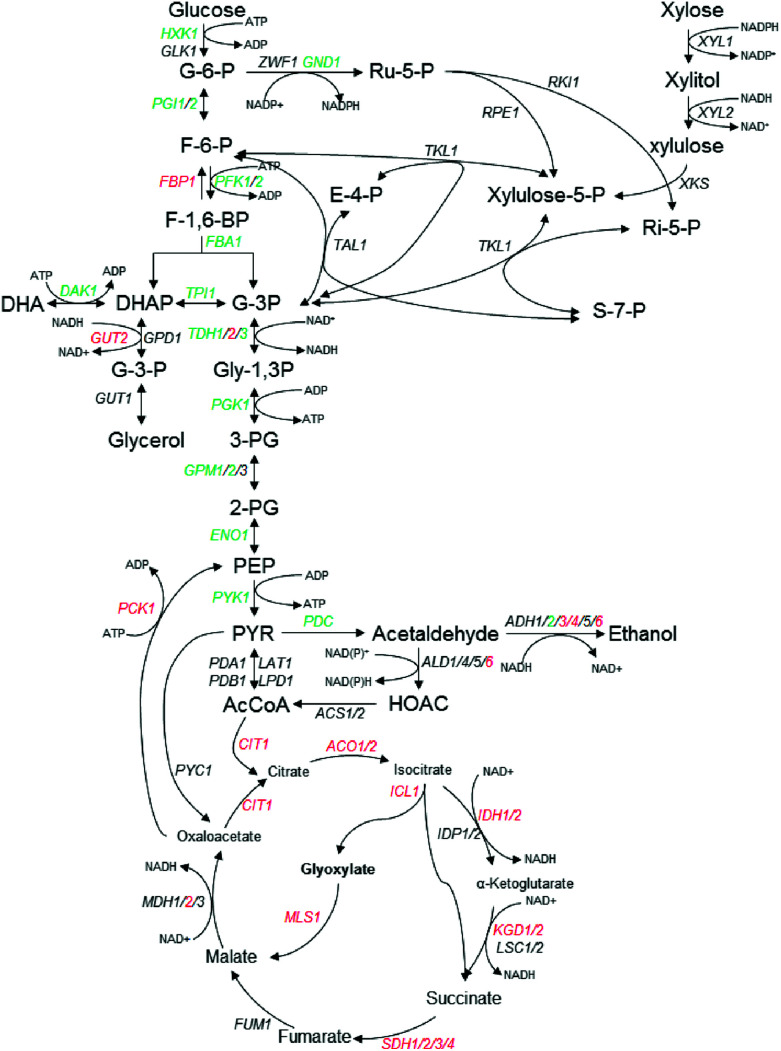
Schematic representation of central carbon metabolism in *K. marxianus* in glycolysis, the pentose phosphate pathway (PPP) and TCA pathway, in response to multiple inhibitors challenges. The fold change (FC) of transcriptional levels with RNA-seq analysis compared with that under no challenge condition was presented by log_2_ FC. Red indicates up-regulated and log_2_ FC ≥ 1, green for down-regulated and log_2_ FC ≤ −1, black indicates no significant change (−1 < log_2_ FC < 1). Further details are given in Table 2.

In this study, most DEGs related to glycolysis/gluconeogenesis and by-products formation were down-regulated except *FBP1*, *GUT2*, *TDH2*, *ADH3*/*4*/*6* and *PCK1* (Table 2 and Fig. 2), which is consistent with relatively slow growth speed with inhibitors stress condition (Fig. 1). In the glycerol formation pathway, *GUT2* encoding glycerol-3-phosphate dehydrogenase was up-regulated with log_2_ FC value of 3.61 under multiple inhibitors condition, while *DAK1* coding for dihydroxyacetone kinase was down-regulated with log_2_ FC value of −2.27(Table 2). Among the most dramatically regulated transcripts are those for glyceraldehyde-3-phosphate dehydrogenase (*TDH1*, *2* and *3*), whereas *TDH1* and *TDH3* were among the most abundant transcripts of all three isoforms and heavily down-regulated with the inhibitors stress, *TDH2* is strongly induced with the same stress. On the other hand, all DEGs related to TCA were up-regulated, and notably, quite a few genes are related to the generation of NADH, such as *IDH1*/*2*, *KGD1*, *2* and *MDH2* (Fig. 2, Table 2).

**Table 2:** Functional categories and fold changes of comparative expression (in form of log_2_ FC) including transcriptional abundance (in form of fpkm) of differentially expressed genes of *K. marxianus* in tolerance to multiple inhibitors^a^

Seq ID	Gene	Description	C fpkm	I fpkm	log_2_ FC(I/C)
**Central carbon metabolism**					
KMAR_60404	*HXK1*	Hexokinase	5322.66	1325.78	−2.01
KMAR_10453	*GND1*	6-Phosphogluconate dehydrogenase	4478.30	1160.97	−1.95
KMAR_10734	*PGI1*	Glucose-6-phosphate isomerase	5935.35	388.91	−3.93
KMAR_10307	*PFK1*	6-Phosphofructokinase subunit alpha	1492.64	89.09	−4.06
KMAR_60457	*PFK2*	6-Phosphofructokinase subunit beta	2477.61	177.67	−3.8
KMAR_60448	*FBP1*	Fructose-1,6-bisphosphatas	102.94	891.30	3.11
KMAR_40392	*FBA1*	Fructose-bisphosphate aldolase	33451.60	1074.41	−4.96
KMAR_40134	*TPI1*	Triosephosphate isomerase	15418.30	667.48	−4.53
KMAR_40225	*TDH1*	Glyceraldehyde-3-phosphate dehydrogenase 1	35695.90	2306.15	−3.95
KMAR_20285	*TDH2*	Glyceraldehyde-3-phosphate dehydrogenase 2	9.34	158.42	4.07
KMAR_80062	*TDH3*	Glyceraldehyde-3-phosphate dehydrogenase 3	98575.50	1358.45	−6.18
KMAR_10522	*PGK1*	Phosphoglycerate kinase	25929.50	1145.68	−4.5
KMAR_20091	*GPM1*	Phosphoglycerate mutase 1	32816.50	662.20	−5.63
KMAR_10274	*GPM2*	Probable phosphoglycerate mutase YOR283W	396.44	89.69	−2.14
KMAR_10447	*ENO1*	Enolase	40558.00	1210.47	−5.07
KMAR_60214	*PYK1*	Pyruvate kinase	14383.50	188.36	−6.25
KMAR_60077	*PDC*	Pyruvate decarboxylase	14769.20	2236.26	−2.72
KMAR_80296	*ADH3*	Alcohol dehydrogenase 3	12.46	58.81	2.23
KMAR_40226	*ADH2*	Alcohol dehydrogenase 2	40021.20	376.60	−6.73
KMAR_20152	*ADH4*	Alcohol dehydrogenase 4	317.36	2881.43	3.18
KMAR_80326	*ADH6*	NADP-dependent alcohol dehydrogenase 6	177.21	2173.89	3.62
KMAR_10714	*ALD6*	Magnesium-activated aldehyde dehydrogenase	738.77	4446.89	2.59
KMAR_50150	*DAK1*	Dihydroxyacetone kinase 1	296.99	61.67	−2.27
KMAR_30696	*GUT2*	Glycerol-3-phosphate dehydrogenase	29.37	360.52	3.61
KMAR_60328	*MAE1*	NAD-dependent malic enzyme	98.33	383.47	1.96
KMAR_20100	*CIT1*	Citrate synthase	774.32	4046.57	2.39
KMAR_30287	*ACO1*	Aconitate hydratase	680.13	3694.41	2.44
KMAR_30288	*ACO2*	Aconitate hydratase	821.58	4035.18	2.3
KMAR_80136	*IDH1*	Isocitrate dehydrogenase [NAD]	171.14	1676.06	3.29
KMAR_20547	*IDH2*	Isocitrate dehydrogenase [NAD]	109.24	1409.28	3.69
KMAR_60528	*KGD1*	2-Oxoglutarate dehydrogenase E1 component	71.21	716.18	3.33
KMAR_50470	*KGD2*	Dihydrolipoyllysine-residue succinyltransferase component of 2-oxoglutarate dehydrogenase complex	160.89	672.17	2.06
KMAR_20443	*SDH1*	Succinate dehydrogenase	155.29	1847.10	3.57
KMAR_20444	*SDH1*	Succinate dehydrogenase	209.65	2222.35	3.41
KMAR_80388	*SDH2*	Succinate dehydrogenase	26.65	396.73	3.89
KMAR_30112	*SDH3*	Succinate dehydrogenase	231.80	3144.05	3.76
KMAR_50521	*SDH4*	Succinate dehydrogenase	112.11	3316.83	4.89
KMAR_60167	*MDH2*	Malate dehydrogenase	648.85	4565.37	2.81
KMAR_30693	*PCK1*	Phosphoenolpyruvate carboxykinase [ATP]	142.00	1386.17	3.29
KMAR_70162	*ICL1*	Isocitrate lyase	4.19	1125.94	8.04
KMAR_60237	*MLS1*	Malate synthase 1	126.57	1108.36	3.13
KMAR_50015	*GDH1*	NADP-specific glutamate dehydrogenase	86.74	389.19	2.16

**Mitochondrial Respiratory chain**					
*NADH dehydrogenase*					
KMAR_10252	*NDI1*	Rotenone-insensitive NADH-ubiquinone oxidoreductase	41.34	446.91	3.43
*Succinate dehydrogenase*					
KMAR_20444	*SDH1*	Succinate dehydrogenase	209.65	2222.35	3.41
KMAR_20443	*SDH1*	Succinate dehydrogenase	155.29	1847.10	3.57
KMAR_80388	*SDH2*	Succinate dehydrogenase	26.65	396.73	3.89
KMAR_30112	*SDH3*	Succinate dehydrogenase	231.80	3144.05	3.76
KMAR_50521	*SDH4*	Succinate dehydrogenase	112.11	3316.83	4.89
*Cytochrome c reductase*					
KMAR_70081	*QCR1*	Cytochrome *b-c1* complex subunit 1	105.22	504.66	2.26
KMAR_40477	*QCR2*	Cytochrome *b-c1* complex subunit 2	99.25	524.10	2.4
KMAR_30195	*QCR9*	c Reductase complex	17.11	92.15	2.42
KMAR_10697	*RIP1*	Cytochrome *b-c1* complex subunit Rieske	146.03	698.92	2.26
KMAR_30247	*CYT1*	Cytochrome *c1*	162.89	783.49	2.27
*F-type ATPase*					
KMAR_70122	*ATP1*	ATP synthase subunit alpha	371.07	1623.39	2.13
KMAR_30175	*ATP16*	ATP synthase subunit delta	151.45	621.88	2.04
KMAR_50127	*ATP14*	ATP synthase subunit H	130.82	521.98	2
V-type ATPase					
KMAR_60174	*ATP6C*	v-Type proton ATPase subunit C	417.08	93.17	−2.16

**ROS detoxification**					
KMAR_70075	*SOD1*	Cu/Zn superoxide dismutase	328.74	2882.30	3.13
KMAR_20527	*SOD2*	Superoxide dismutase [Mn]	276.07	3969.21	3.85
KMAR_40107		Hyperthetical protein, cell surface superoxide dismutase [Cu–Zn]	0.50	32.35	5.75
KMAR_80342	*PRX1*	Mitochondrial peroxiredoxin PRX1	170.45	1580.35	3.21
KMAR_40185	*AHP1*	Peroxiredoxin type-2	6824.27	853.08	−3
KMAR_50400	*CTT1*	Catalase T	2052.19	44.59	−5.52

**MSN2/4 mediated STRE related DEGs**					
KMAR_60404	*HXK1*	Hexokinase	5322.66	1325.78	−2.01
KMAR_20247	*GPH*	Glycogen phosphorylase	860.61	204.77	−2.07
KMAR_80350	*SSA3*	Heat shock protein	936.81	5531.12	2.56
KMAR_50400	*CTT1*	Catalase T	2052.19	44.59	−5.52
KMAR_20527	*SOD2*	Superoxide dismutase [Mn]	276.07	3969.21	3.85
KMAR_40225	*TDH1*	Glyceraldehyde-3-phosphate dehydrogenase 1	35695.90	2306.15	−3.95
KMAR_20285	*TDH2*	Glyceraldehyde-3-phosphate dehydrogenase 2	9.34	158.42	4.07
KMAR_80062	*TDH3*	Glyceraldehyde-3-phosphate dehydrogenase 3	98575.50	1358.45	−6.18
KMAR_30091	*PGM*	Phosphoglucomutase-2	1598.30	301.41	−2.41
KMAR_40137	*HSP26*	Heat shock protein	920.24	63986.60	6.12
KMAR_80025	*HSP31*	Probable chaperone protein HSP31	43.91	2233.33	5.67
KMAR_10714	*ALD6*	Magnesium-activated aldehyde dehydrogenase	738.77	4446.89	2.59
KMAR_60167	*MDH2*	Malate dehydrogenase	648.85	4565.37	2.81

**Fatty acid and ergosterol metabolism**					
KMAR_10220	*OLE1*	Acyl-CoA desaturase 1	8230.00	1131.74	−2.86
KMAR_10557	*SCS7*	Inositolphosphorylceramide-B C-26 hydroxylase	1347.86	246.20	−2.45
KMAR_70200	*FAS2*	Fatty acid synthase subunit alpha	1111.69	258.89	−2.1
KMAR_20691	*DUG3*	Probable glutamine amidotransferase DUG3	826.29	207.67	−1.99
KMAR_50026	*LipA*	Lipoyl synthase	65.99	262.77	1.99
KMAR_50263	*ERG25*	c-4 Methylsterol oxidase	1518.63	334.97	−2.18
KMAR_80146	*LTA4H*	Leukotriene A-4 hydrolase	376.46	75.65	−2.31
KMAR_30191	*ERG1*	Squalene monooxygenase	447.57	37.96	−3.56
KMAR_60441	*ATH1*	Vacuolar acid trehalase	552.26	101.55	−2.44
KMAR_10355	*ERG20*	Farnesyl pyrophosphate synthetase	1735.84	412.86	−2.07

**Alanine, aspartate and glutamate metabolism**					
KMAR_20293	*AGX1*	Alanine-glyoxylate aminotransferase 1	26.1494	226.387	3.11
KMAR_40206	*UGA1*	4-Aminobutyrate aminotransferase	21.6204	268.061	3.63
KMAR_50015	*GDH1*	NADP-specific glutamate dehydrogenase 2	86.7376	389.188	2.16
KMAR_50578	*ADSS*	Adenylosuccinate synthetase	1366.54	356.931	−1.94
KMAR_70254	*ASN1*	Asparagine synthetase 1 [glutamine-hydrolyzing]	1302.34	312.433	−2.06

**Vitamin B6 and B1 metabolism**					
KMAR_30698		Probable pyridoxine biosynthesis protein SNZ3	3057.97	293.06	−3.38
KMAR_30699		Probable pyridoxal 5'-phosphate synthase SNO3	373.66	34.44	−3.44
KMAR_30041		Phosphomethylpyrimidine kinase THI20	99.31	9.26	−3.41
KMAR_20540		Thiamine pyrophosphokinase	162.39	32.08	−2.34
KMAR_40549	*THI6*	Thiamine biosynthetic bifunctional enzyme	69.55	15.94	−2.12
KMAR_30339		Putative pyridoxal reductase	145.08	1063.71	2.87

**NAD** ^**+**^ **synthesis**					
KMAR_30654	*SDT1*	Suppressor of disruption of TFIIS	15.53	78.23	2.33

**Transcription factors**					
KMAR_30570	*OAF1*	Oleate-activated transcription factor 1	0.99	9.04	3.07
KMAR_50272	*MTF1*	Mitochondrial transcription factor 1	2.34	18.82	2.96
KMAR_30474	*HCM1*	Forkhead transcription factor HCM1	7.94	38.15	2.25
KMAR_30246	*YNG1*	Protein YNG1	5.68	27.32	2.25
KMAR_60382	*MET32*	Transcriptional regulator MET32	15.42	72.85	2.23
KMAR_50274	*SNF2*	Transcription regulatory protein SNF2	29.48	127.94	2.11
KMAR_40216	*GCR2*	Hypothetical glycolytic genes transcriptional activator GCR2	113.63	23.56	−2.27
KMAR_40526	*ASH1*	Transcriptional regulatory protein ASH1	34.47	6.47	−2.40
KMAR_70129	*MED19*	Mediator of RNA polymerase II transcription subunit 19	680.56	108.18	−2.65
KMAR_10730	*GCR1*	Glycolytic genes transcriptional activator GCR1	33.84	5.22	−2.67
KMAR_60223	*TFC7*	Transcription factor C subunit 7	851.59	102.43	−3.05
KMAR_40048	*TFIIF2*	Transcription initiation factor IIF subunit beta	61.40	319.74	2.38

**Transporters**					
***MFS protein***					
*Sugar transporter*					
KMAR_60316		Uncharacterized transporter YHL008C	11.99	156.02	3.69
KMAR_80370	*HXT14*	Hexose transporter HXT14	1.28	11.64	3.09
KMAR_30579	*STL1*	Sugar transporter STL1	8.32	60.20	2.84
KMAR_80266	*ITR2*	Myo-inositol transporter 2	19.96	126.41	2.66
KMAR_50347	*RAG1*	Low-affinity glucose transporter	16.62	78.51	2.23
KMAR_20602		Putative polyol transporter 2	1.77	15.93	3.1
KMAR_70126		Conserved hypothetical membrane protein	10.97	82.77	2.9
KMAR_10531		High-affinity glucose transporter	23.83	153.39	2.68
KMAR_50344	*HXT2*	Hexose transporter 2	205.68	49.50	−2.05
KMAR_10529		High-affinity glucose transporter	18.87	3.39	−2.44
*Amino acid transporter*					
KMAR_40029	*YCT1*	High affinity cysteine transporter	5.42	61.10	3.47
KMAR_10514	*TAT2*	Tryptophan permease	101.61	13.66	−2.89
KMAR_10360	*GAP1*	General amino-acid permease GAP1	56.76	9.82	−2.52
*Multidrug permease*					
KMAR_50130	*FNX1*	Multidrug resistance protein fnx1	25.70	106.64	2.05
KMAR_80409	*ATR1*	Aminotriazole resistance protein	11.22	71.74	2.67
*Allantoate permease*					
KMAR_60406	*DAL5*	Allantoate permease	3.06	24.03	2.93
KMAR_10004	*SEO1*	Probable transporter SEO1	1.14	8.43	2.78
*Others*					
KMAR_40093	*ARN2*	Siderophore iron transporter ARN2	10.77	397.56	5.19
KMAR_10790	*SIT1*	Siderophore iron transporter 3	15.34	153.18	3.31
KMAR_20248	*JEN2*	Putative sialic acid transporter	37.42	307.39	3.03
KMAR_40425		Uncharacterized Polyamine transporter 4	33.54	264.91	2.98
KMAR_60075	*JEN1*	Carboxylic acid transporter protein homolog	198.52	1194.02	2.59
KMAR_30642		Probable metabolite transport protein C1271.09	12.14	68.78	2.49
KMAR_10458	*TNA1*	High-affinity nicotinic acid transporter	78.73	6.17	−3.65
KMAR_10759		Uncharacterized transporter YBR180W	122.32	8.89	−3.77
KMAR_20313	*MCH5*	Riboflavin transporter MCH5	316.12	78.57	−2.01

**ABC transporter**					
KMAR_30337	*PDR12*	ATP-dependent permease PDR12	34.49	815.27	4.56
KMAR_40188	*YCF1*	Metal resistance protein YCF1	8.40	32.14	1.92

**Sulfate permease**					
KMAR_40156	*SUL2*	Sulfate permease 2	5.77	60.90	3.38

**Ammonia permease**					
KMAR_70262	*MEP3*	Ammonium transporter MEP3	8.25	36.60	2.14

**Purine/cytosine permease**					
KMAR_70169		Purine-cytosine permease FCY2	542.96	135.11	−2.01
KMAR_10802		Purine-cytosine permease FCY2	10.84	42.91	1.98

**oligopeptide transporter**					
KMAR_80400		Uncharacterized oligopeptide transporter C1840.12	16.36	156.57	3.25
KMAR_20003	*OPT1*	Oligopeptide transporter 1	45.37	7.42	−2.6

**Transporters with no MFS**					
KMAR_70277	*CTR1*	Copper transport protein CTR1	2291.30	328.30	−2.8
KMAR_40340		Cystine transporter	18.76	169.50	3.17
KMAR_20004		Probable urea active transporter 1	6.07	26.49	2.11
KMAR_30588	*FTR1*	Plasma membrane iron permease	149.15	712.78	2.26
KMAR_70319	*PET9*	Mitochondrial ADP, ATP carrier protein	410.83	2913.82	2.83
KMAR_30323	*AQY1*	Aquaporin-1	248.01	53.00	−2.22
KMAR_40422	*FSF1*	Probable mitochondrial transport protein FSF1	400.58	75.03	−2.42
KMAR_60332	*CTP1*	Tricarboxylate transport protein	193.04	41.79	−2.2
KMAR_50593	*FET4*	Low-affinity Fe(2+) transport protein	116.55	6.48	−4.15

a Only differentially expressed genes were presented in the table. I and C represent samples of yeast grown on medium with or without multiple inhibitors in YPD under aerobic condition.

It was noticeable that the isoforms of ADH were dramatically regulated with the multiple inhibitors stress condition. As shown in Table 2, the transcript of *ADH2* (KMAR_40226), *ADH4* (KMAR_20152) and *ADH6* (KMAR_80326) was the three highest abundant isoforms. Under the multiple inhibitors stress, *ADH4* and *ADH6* were up-regulated with log_2_ FC value of 3.18 and 3.62, respectively. *ADH3*, another ADH isoform which encodes an ethanol–acetaldehyde redox shuttle involved in the transfer of redox equivalents from the mitochondria to the cytosol, was up-regulated with log_2_ FC value of 2.23 (Fig. 2 and Table 2), consistent with previous studies that *ADH3*-disrupted *K. marxianus* was more sensitive to the reactive oxygen species and the null mutant of *ADH6* was hypersensitive to vanillin, a major phenolic aldehyde compounds derived from lignocellulosic biomass, in *S. cerevisiae*.^18,27^ On the other hand, *ADH2* was down-regulated with log_2_ FC value of −6.73. Unlike other ADHs, ADH2 catalyzes the reaction of ethanol to acetaldehyde and is repressed in the presence of glucose, and the repressed expression in our study might be explained that the addition of furfural inhibited the glucose consumption and led to higher glucose concentrations and this in turn repressed the expression of *ADH2*.^28^

### DEGs related to mitochondrial respiratory chain and ATPases

The mitochondrial respiratory chain, which forms membrane potentials to produce ATP by ATPase, consists of vital components to transfer electrons, and those electron carriers function in the form of multienzyme complexes. As shown in Table 2, under the stress of the multiple inhibitors, *NDI1* coding rotenone-insensitive NADH-ubiquinone oxidoreductase, and all of the *SDHs* coding rotenone-insensitive NADH-ubiquinone oxidoreductases were dramatically up-regulated more than 8-fold (log_2_ FC > 3). And almost half genes of those coding cytochrome *b-c1* complex units were up-regulated (Table 2). However, to those coding core subunits of cytochrome *c* oxidase, there was no quite difference with the stress of the mixed inhibitors (data not shown). Previous report showed that mutation in SDH increased ROS production to nuclear and mitochondrial genomic instability.^29^ Our findings indicated that to some extent SDH-*bc*1 complex-cytochrome *c* peroxidase played a role in scavenging ROS released from the tolerance to multiple inhibitors in *K. marxianus*.

F_1_F_0_ ATP synthase is a large, evolutionarily conserved enzyme complex required for ATP synthesis. Among vast genes encoding subunits of F_1_F_0_ ATP synthase complex (F-type ATPase), only *ATP1*, *ATP14* and *ATP16* which encoding alpha subunit of the F_1_ sector, subunit h of the F_0_ sector, and delta subunit of the central stalk of mitochondrial F_1_F_0_ ATP synthase, respectively, were up-regulated more than 4 fold (log_2_ FC ≥ 2) under multiple inhibitors (Table 2), suggesting that the energy production was important to the tolerance to inhibitors stress.

V-ATPase maintains the acidity of the vacuole and generates the electrogenic potential that is used to drive the accumulation of ions and small molecules, amino acids and metabolites. V-ATPase-depleting mutants exhibited sensitivity to the acids.^30^ Interestingly, novel roles of V-ATPase in the regulation of cellular receptors and their trafficking *via* endocytotic and exocytotic pathways were recently uncovered.^31^ Also, defects in acidification, through defects in the vacuolar H^+^-ATPase, will lead to defective assembly of the high affinity iron transport system.^32^ In this study, *ATP6c* coding v-type proton ATPase subunit c was down-regulated under multiple inhibitors stress, meanwhile, iron transporters coding genes such as *ARN2*, *SIT1* were up-regulated (Table 2).

### DEGs involved in genes related to ROS detoxification

Reactive oxygen species (ROS) are a group of molecules derived from molecular oxygen and have toxic effects that can damage a wide variety of cellular components resulting in lipid peroxidation, protein oxidation, and genetic damage through the modification of DNA. Inhibitors like acetic acid, furfural, and phenols have been reported to be related to the redox state inside cells, inducing reactive oxygen species (ROS) generation.^33–35^ Genes related to ROS detoxification including those coding for superoxide dismutases (SODs) and their chaperones, catalases and peroxidases, glutathione and thioredoxin systems. These proteins remove excess ROS such as ˙OH, H_2_O_2_, and O_2_˙^−^*etc.* by participating in oxidation–reduction reactions to maintain normal cell metabolism and to ensure a high rate of cell viabilities by their activated dimers. Interestingly, only several oxidative stress-response genes were found to be up- or down-regulated under the mixed inhibitors treatment (Table 2). As shown in Table 2, *SOD1* and *SOD2*, coding cytosol Cu/Zn superoxide dismutase and mitochondrial superoxide dismutase [Mn] respectively, were up-regulated under the stress of multiple inhibitors, suggesting that SODs played an important role in multiple inhibitors tolerance in *K. marxianus*. *CTA1* and *CTT1*, corresponding to two isoforms of catalase with different sub-cellular locations, peroxisomal–mitochondrial matrices and cytosol, respectively,^36^ while *CTT1* was down-regulated significantly to −5.52 of the log_2_ FC under the stress of inhibitors (Table 2), *CTA1* had no significant change (data not shown). As to those genes encoding thioredoxin peroxidases, *PRX1* was up-regulated with log_2_ FC value of 3.21 while *AHP1* was down-regulated with log_2_ FC value of −3 under the stress of mixed inhibitors (Table 2). It was reported that expression of *TSA1* of *K. marxianus* which encoding peroxiredoxin TSA1 enhanced the tolerance to a mixture of formic acid, acetic acid and furfural in *S. cerevisiae*.^37^ In this study, however, there was no significant change with *TSA1* (data not shown), indicating the different role of this TSA1 in *K. marxianus* from that in *S. cerevisiae*.

The essential coenzymes nicotinamide adenine dinucleotides, NAD(P)^+^ and NAD(P)H, participate in key redox reactions and contribute to maintaining cell fitness and genome stability.^38^ Those genes such as *ADH3*, *ALD6*, *IDH1*/*2*, *GDH1* and *NDI1 etc.* coding for NAD(P)H/NAD(P)^+^ shuttle systems which play a key role in the maintenance of the mitochondrial redox balance by redox transformation from NAD(P)^+^ to NAD(P)H were up-regulated in our RNA-seq result (Table 2).

**Fig. 3:**
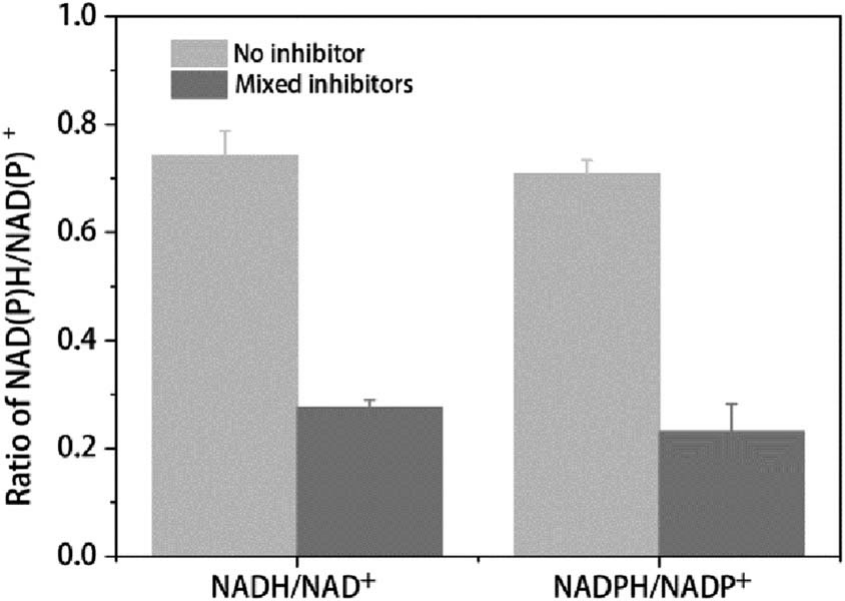
Ratio of intracellular NAD(P)H/NAD(P)^+^ with or without mixed inhibitors. The error bars represent the standard deviation calculated from triplicate experiments.

The ratio between reduced and oxidized co-factors is thought to play a major role in metabolism since several enzymes are regulated by this ratio.^39^ In the present study the NADH/NAD^+^ and NADPH/NADP^+^ ratio were used to determine the change of redox balance. As shown in Table 3 and Fig. 3, with 2 h multiple inhibitors treatment, the concentration of NAD^+^ was dramatically increased from 394.64 nmol g^−1^ DCW to 887.63 nmol g^−1^ DCW, while the concentration of NADH was only a little less than that of no stress, leading to the ratio of NADH/NAD^+^ decreased from 0.74 to 0.28 (Table 3 and Fig. 3). The concentration of NADH and NAD^+^ pool was increased from 686.96 nmol g^−1^ DCW to 1132.14 nmol g^−1^ DCW. On the other hand, with the multiple inhibitors stress, the concentration of NADP^+^ was increased from 37.88 nmol g^−1^ DCW to 58.28 nmol g^−1^ DCW, while the concentration of NADPH was decreased from 26.84 nmol g^−1^ DCW to 13.40 nmol g^−1^ DCW, leading to the ratio of NADH/NAD^+^ decreased from 0.71 to 0.23 (Table 3 and Fig. 3.). The concentration of NADPH and NADP^+^ pool was increased from 64.72 nmol g^−1^ DCW to 71.68 nmol g^−1^ DCW, not changed so much like NADH + NAD^+^ pool (Table 3). As a result, with the multiple inhibitors stress, the concentration of total coenzymes was dramatically increased from 751.69 nmol g^−1^ DCW to 1203.81 nmol g^−1^ DCW (Table 3). Consistently, *SDT1* encoding suppressor of disruption of TFIIS which was reported to be responsible for production of precursors in NAD^+^ synthesis in cells,^40^ was up-regulated in our study (Table 2).

**Table 3:** Levels of intracellular coenzymes with or without multiple inhibitors

	NAD^+^ (nmol g^−1^ DCW)	NADH (nmol g^−1^ DCW)	NADP^+^ (nmol g^−1^ DCW)	NADPH (nmol g^−1^ DCW)	NADH/NAD^+^	NADPH/NADP^+^	NADH + NAD^+^ (nmol g^−1^ DCW)	NADPH + NADP^+^ (nmol g^−1^ DCW)	Total coenzymes (nmol g^−1^ DCW)
Control	394.64 ± 19.47	292.33 ± 15.68	37.88 ± 2.19	26.84 ± 1.65	0.74	0.71	686.96	64.72	751.69
Mixed inhibitors	887.63 ± 24.82	244.51 ± 7.66	58.28 ± 1.43	13.40 ± 2.79	0.28	0.23	1132.14	71.68	1203.81

### MSN2/4 mediated STRE related DEGs

MSN2/4 regulated the expression of a wide variety of genes in response to multiple types of stress in *S. cerevisiae*.^41^Table 2 lists DEGs which are reported to be controlled *via* the stress responsive element (STRE) in *S. cerevisiae*.^41,42^ As shown in Table 2, quite a few stress-responsive genes were down-regulated under the stress of multiple inhibitors, such as *HXK1*, *GPH*, *TDH1*, *TDH3*, *PGM*, *CTT1 etc.*, while there were also some genes up-regulated such as *SSA3*, *SOD2*, *TDH2*, *HSP26*, *HSP31*, *ALD*, *MDH2 etc.* Our RNA-seq results showed that there was only MSN2 in *K. marxianus* and with no significant change under the multiple inhibitors condition (data not shown), indicating that this MSN2 might be different to that in *S. cerevisiae*. Due to the few study of this protein in *K. marxianus*, the specific function of MSN2 needs to be further investigated.

### DEGs related to fatty acid and ergosterol metabolism

Ergosterol and fatty acids are considered as two critical membrane components associated with tolerance to multiple stresses.^43,44^ As shown in Table 2, there were 7 DEGs with related to fatty acid metabolic process, and 3 DEGs with related to the ergosterol biosynthetic process, and almost all of them were down-regulated except *LipA* (*KMAR_*50026) coding lipoyl synthase. Among those down-regulated genes, *OLE1* encodes acyl-CoA desaturase 1 involved in desaturation of fatty acid and with greatest transcriptional abundance (Table 2).

### DEGs related to alanine, aspartate and glutamate metabolism, vitamin B1 and B6 metabolism

In our study, *AGX1* encoding alanine-glyoxylate aminotransferase 1 related to alanine synthesis, *UGA1* encoding 4-aminobutyrate aminotransferase and *GDH1* encoding NADP-specific glutamate dehydrogenase 2 which are related to glutamate synthesis, respectively, were up-regulated with the multiple inhibitors stress condition, while *ADSS* encoding adenylosuccinate synthetase and *ASN1* encoding asparagine synthetase 1 related to aspartate synthesis were down-regulated with the same stress condition (Table 2).

As to vitamin B6 and B1 metabolism, all the DEGs related to this category were down-regulated except one gene encoding a putative pyridoxal reductase with the multiple inhibitors stress (Table 2). Interestingly, though genes encoding probable pyridoxine biosynthesis protein SNZ3 and probable pyridoxal 5′-phosphate synthase SNO3 were dramatically down-regulated (Table 2), in our result, however, there were no *SNZ1* and *SNO1* corresponding to the counterparts of *S. cerevisiae* in *K. marxianus*, which suggests that SNZ3 and SNO3 of *K. marxianus* might be quite different from those in *S. cerevisiae*.

### DEGs related to transcription factors

Transcription factors (TF) play an important role in regulatory mechanisms underlying various stresses resistance mechanisms. As shown in Table 2, DEGs related to TF are widely distributed in the regulatory network for diverse biological processes, including carbohydrate metabolism (*GCR1*, *GCR2*), sulfur amino acids metabolism (*MET32*), lipid metabolism (*OAF1*), cell proliferation (*HCM1*), and transcriptional process (*YNG1*, *TFC7*, *MTF1*, *MED19*, *TFIIF2*) *etc.* of those TFs with increased transcriptional expression under inhibitors stress condition, HCM1, as a transcription factor involved in cell cycle regulation, is also involved in promoting mitochondrial biogenesis and stress resistance in *S. cerevisiae*;^45^ OAF1 was reported to regulate genes involved in beta-oxidation of fatty acids, peroxisome organization and biogenesis, activating transcription in the presence of oleate,^46^ though the transcript abundance in this study is very low. As to the carbohydrate metabolism related TF genes, *GCR1* and *GCR2* encoding glycolytic genes transcriptional activator GCR1 and GCR2, respectively, were down-regulated with multiple inhibitors stress, and this was consistent with a previous report that GCR1 and GCR2 mutants showed lower glycolytic activities and enhanced the expression of TCA and respiratory genes to produce more energy.^47^

We also noticed that several DEGs related to the transcriptional factors that are directly involved in the transcriptional process were regulated with the multiple inhibitors stress. *TFIIF2* encoding transcription initiation factor IIF subunit beta, *MTF1* encoding a mitochondrial transcriptional factor that confers selective promoter recognition on the core subunit of the yeast mitochondrial RNA polymerase, and *YNG1* encoding a component of the NuA3 histone acetyltransferase complex that post-translationally modifies histones,^48^ were up-regulated, while *TFC7* encoding a component of the initiation complex which functioned in RNA polymerase III recruitment and *MED19* encoding a subunit of mediator were down-regulated with multiple inhibitors stress (Table 2). Mediator binds transcription activation domains and Pol II, allowing activator-dependent Pol II recruitment.^49,50^ These results indicated that the processes of transcription initiation, transcription activation, the promoter recognition were selectively regulated by the multiple inhibitors stress.

### DEGs related to transporters and the transcriptional response with individual inhibitor stress

**Fig. 4:**
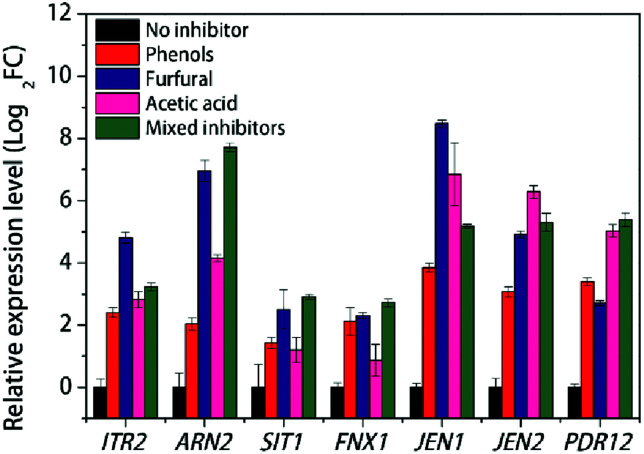
RT-PCR results of various transporters with individual inhibitor stress condition in *K. marxianus*.

Transporters play key roles in the response of the fermentation inhibitors. As an important component of transporters, major facilitator superfamily (MFS) is a large and diverse group of secondary transporters that includes uniporters, symporters, and antiporters.^51^ As shown in Table 2, 26 genes containing MFS domain were identified to have different expression levels, including 19 up-regulated genes and 7 down-regulated genes. Under the stress of multiple inhibitors, most sugar transporters DEGs were up-regulated except *HXT2* (KMAR_50344) and KMAR_10529 coding for hexose transporter 2 and high-affinity glucose transporter respectively. *ITR2* encoding myo-inositol transporter 2 was up-regulated with log_2_ FC value of 2.66, and further RT-PCR results showed that *ITR2* was up-regulated dramatically with furfural stress, up to 4.8 of log_2_ FC value compared with that with no stress (Fig. 4). Previous report showed that myo-inositol improved tolerance of *S. cerevisiae* to the mixture of furfural, acetic acid and phenol, and deletion of gene in myo-inositol synthesis weakened strain tolerance against this stress.^52^ Our result further indicated that ITR2 may play an important role against furfural in lignocellulose-derived inhibitors stress.

As to the amino acid transporters, *TAT2* (KMAR_10514) encoding tryptophan permease and *GAP1* encoding general amino-acid permease were down-regulated with log_2_ FC value of −2.89 and −2.52, respectively, under the stress of mixed inhibitors. Tryptophan can be converted to quinolinic acid (QA), an important precursor in NAD + synthesis.^53^ On the other hand, *YCT1* encoding high affinity cysteine transporter and KMAR_40340 encoding cystine transporter were up-regulated about 8-fold than that with no stress condition (Table 2).

Under the stress of multiple inhibitors, *TNA1*, encoding high-affinity nicotinic acid transporter which was essential for the NAD^+^ homeostasis,^54^ was down-regulated, while *DAL5* encoding an allantoate and ureidosuccinate permease subjected to nitrogen catabolite repression^55,56^ and *SUL2* encoding sulfate permease 2 were up-regulated, but the transcript abundance was too low (Table 2). In *S. cerevisiae*, it was observed that the genes involved in sulfur metabolism are mainly regulated by the cellular cysteine pool.^57^

The ARN family encodes proteins involved in the uptake of siderophore-iron chelates. Genome-wide analysis showed that the acidic condition affects metal metabolism.^30^ From our RNA-seq results, *ARN2* and *SIT1* were significantly up-regulated with log_2_ FC value of 5.19 and 3.31, respectively, under the stress of mixed inhibitors (Table 2). *FTR1* encoding plasma membrane iron permease was also up-regulated, though *FET4* encoding low-affinity Fe^2+^ transport protein was down-regulated. Meanwhile, a gene *FSF1* encoding a probable mitochondrial transporter which was reported to be necessary to maintain the homeostasis of iron,^58^ was down-regulated with multiple inhibitors condition (Table 2). ARN2 was found to be induced under the acid adaptation and acid affects metal metabolism.^30^ In our individual inhibitor stress experiment, however, though *ARN2* was induced under the acetic acid condition, the most-enhanced expression was with furfural stress, and so was that of *SIT1* (Fig. 4), indicating that furfural may affect iron transportation more than acidic condition in *K. marxianus*. High affinity copper transporter coding gene, *CTR1*, was repressed with the multiple inhibitors stress (Table 2). Interestingly, low affinity copper uptake can be mediated by FET4, which was also low affinity iron transporter,^59,60^ and the coding gene *FET4* was also repressed in this study (Table 2), indicating that the multiple inhibitors stress inhibited the copper uptake.

In our study, *MEP3* encoding ammonium transporter MEP3 was up-regulated, while *OPT1* encoding oligopeptide transporter 1 and *CTP1* encoding a tricarboxylate transport protein were down-regulated with the multiple stress (Table 2).

Under the stress of multiple inhibitors, *PET9* encoding a mitochondrial ADP, ATP carrier protein was dramatically up-regulated, which was consistent with the up-regulation of those genes coding for ATP synthase (Table 2).

Efflux system of living cells is an efficient mechanism for detoxification of external toxic compounds and internal damaging intermediates. Two multidrug permease gene, *ATR1* encoding aminotriazole resistance protein and *FNX1* encoding multidrug resistance protein were up-regulated under the mixed fermentation inhibitors (Table 2). *FNX1* also showed increased transcriptional expression under furfural or phenols stress (Fig. 4). These results were consistent to previous reports that *ATR1* deletion mutant *S. cerevisiae* showed increased sensitivity to lignocellulosic inhibitors and *FNX1* mutant *S. pombe* presented impaired uptake of vacuolar amino acid.^61,62^

In addition, KMAR_40425 encoding an uncharacterized polyamine transporter 4 was up-regulated under multiple inhibitors stress (Table 2), consistent to a recent report that higher spermidine was able to enhance tolerance of *S. cerevisiae* against lignocellulose-derived inhibitors.^33^

Carboxylic acid transporter protein JEN1 was found to be involved in the acids efflux and the transport of the substrate is bidirectional.^63–65^ Our RT-PCR results showed that *JEN1* was significantly up-regulated with log_2_ FC value of 8.47, 6.85 and 3.84, under the furfural, acetic acid and phenols stress respectively, compared with no stress condition (Fig. 4). Meanwhile, another gene *JEN2* encoding putative sialic acid transporter with 34.7% identity with JEN1 of *S. cerevisiae* and 74.3% identity with JEN2 of *Kluyveromyces lactis*, was up-regulated with log_2_ FC value of 4.91, 6.28 and 3.07, under the furfural, acetic acid and phenols stress respectively, compared with no stress condition (Fig. 4). Both RNA-seq and RT-PCR results showed that these two genes were up-regulated under the multiple inhibitors stress (Table 2 and Fig. 4). This suggests that *JEN1* and *JEN2* respond with different stress and play an important role against the mixed inhibitors stress.

PDR12, an ATP-binding cassette (ABC) transporter and a member of the Pleiotropic Drug Resistance (PDR) family, was demonstrated to be essential to the acquisition of tolerance to weak acid stress, being involved in the extrusion of the carboxylate anions and participating in cellular detoxification.^66^ Our RT-PCR results also showed that *PDR12* was induced with log_2_ FC value of 5.03 in respond to acetic acid stress compared with no stress condition, the most up-regulated among three stress conditions (Fig. 4). This suggests that PDR12 be an interesting protein especially against acid stress. Another ABC transporter gene *YCF1*, encoding metal resistance protein YCF1 which was reported to function in the detoxification of furfural and/or HMF^67^ and mediated transport of GSH-conjugated metals for metal tolerance,^68^ was also up-regulated under mixed inhibitors stress (Table 2).

## Discussion

Exploring the toxicity of lignocellulose-derived inhibitors to yeasts and developing the excellent strains with enhanced tolerance are becoming a more critical component of producing chemical products from lignocellulosic materials. Transcriptomic data obtained from inhibitors tolerance experiments is an extremely important step in the construction of large scale, system-based models that can be used to predict the cellular response to this stress. The integration of this data with pathway information is crucial to improve the accuracy in the prediction capabilities of the models. In the case of unconventional thermotolerant yeast *K. marxianus*, however, there were only several reports of transcriptome analysis on yeast responses from different carbon source including glucose, inulin and xylose, or the tolerance to high temperature or ethanol stress.^18,69,70,71^ To the best of our knowledge, genome-wide transcription analysis under the multiple inhibitors stress condition with elevated temperature (42 °C) has not been reported for this yeast.

Carbon central metabolism plays an important role in carbon source and energy production to yeast cells. From our results, differentially expressed genes related to the carbon central metabolism were selectively regulated by multiple inhibitors stress. Though previous report showed that the genes and proteins associated with glycolysis were over-expressed under acetic acid stress,^72,73^ we noticed that DEGs related to the glycolysis were depressed in response to the multiple inhibitors (Table 2, Fig. 2), while those related to TCA and a gluconeogenesis specific gene *FBP1* were up-regulated, and consistently, most DEGs encoding the respiratory chain component functioned in the oxidative phosphorylation in mitochondria were up-regulated (Table 2 and Fig. 2), together with the up-regulation of mitochondrial ADP/ATP carrier gene *PET9*, suggesting that inhibitors stimulate cells to produce more ATP. Cells need to choose the most efficient route to generate energy or reduce ATP consumption to maintain energy reserves under environmental stress condition. We speculate that cells choose to slow down the metabolic flux in glycolysis pathway while turn to enhance TCA cycle to obtain more ATP production and more NADH, since detoxification of furfural or phenolic compounds is an energy-consuming process. Coincidently, the carbohydrate metabolism related TF genes *GCR1* and *GCR2* were also down-regulated with multiple inhibitors stress (Table 2). GCR1 and GCR2 mutants were reported to show lower glycolytic activities and enhanced the expression of TCA and respiratory genes to produce more energy,^47^ in addition, overexpression of GCR1 increased transcription levels of *HXT1* and ribosomal protein genes in *S. cerevisiae*.^74^ Combined with our results, these studies indicated that in *K. marxianus* GCR1 and GCR2 may play a role with tolerance to the hydrolysates inhibitors by regulating carbon central metabolism process to produce more energy.

On the other hand, as a protective mechanism responding to environmental stress, glycerol played a key role in keeping high cell viabilities during ethanol fermentation. In accordance with this, up-regulation of GUT2 and down-regulation of DAK1 in favor of the glycerol formation pathway was observed (Table 2).

As three main lignocellulose-derived inhibitors, acetic acid affects cell metabolism and stabilities of proteins by a drop in intracellular pH and membrane potential, furfural inhibits glycolytic and fermentative enzymes essential to central metabolic pathways, and phenolic compounds alter the permeability of biological membranes and caused irreversible damages to the cells.^75^ All these inhibitors have been reported to be related to the redox state inside cells inducing ROS generation.^33–35^ Furfural and HMF were reported to inhibit alcohol dehydrogenase (ADH), pyruvate dehydrogenase (PDH), aldehyde dehydrogenase (ALDH), hexokinase (HXK) and glyceraldehyde-3-phosphate dehydrogenase (GPDH) in *S. cerevisiae*,^75^ in our study, however, at least at the transcriptional level, only *HXK*, *ADH2* and *TDH1*/*3* were down-regulated, *ADH3*/*4*/*6* and *ALD6* were up-regulated, and there was no obvious change on genes coding for pyruvate dehydrogenase (Table 2 and Fig. 2).

Previous study reported that NADPH-dependent oxidoreductases comprise the main resistance mechanism for high concentrations of furfural.^76^ Expression of some oxidoreductases could enhance the tolerance of cells to furfural, acetic acid and phenolic compounds in lignocellulosic hydrolysates, and intracellular ROS in cells with an increased tolerance has been reported to be decreased.^37,77,78^ In our study, in response to multiple inhibitors stress, the transcripts for the genes encoding known NAD(P)H/NAD(P)^+^ shuttle systems, including *ADH3*/*4*/*6*, *ALD6*, *TDH2*, *GUT2*, *IDH1*/*2*, *GDH1* and *NDI1* showed high levels of enhanced expressions, and transcripts for enzymes involved in the malate-oxaloacetate shuttle or malate-pyruvate shuttle, encoded by *MDH2* and *MAE1*, were also induced (Fig. 2, Table 2). Previous report showed that MDH could be regarded as a transhydrogenase-like shunt, which regulated the redox state in *S. cerevisiae*.^79^

ROS overproduction in response to the inhibitors is another reason for redox imbalance in yeast. ROS scavenging proteins remove excess ROS such as ˙OH, H_2_O_2_, and O_2_˙^−^*etc.* generated from the multiple inhibitors by participating in oxidation–reduction reactions and this requires the reducing power. Detoxification of lignocellulosic inhibitors like furfural, HMF or phenolic compounds is a process of converting them into less toxic corresponding alcohols in NAD(P)H-dependent reduction,^5,80^ which requires the supply of sufficient amounts of the involved co-enzymes. This was consistent to the decrease of NAD(P)H/NAD(P)^+^ ratio in our study and others report.^28^ In this study, the total amount of NAD^+^ and NADH increased nearly one fold when the strain exposed to inhibitors (Table 3), whereas the total amount of NAD^+^ and NADH was decreased in the case of *S. cerevisiae*.^28^ One possible reason of the increased intracellular concentration of NAD^+^ might be that the NAD^+^ synthesis was increased under the multiple stresses, based on the up-regulation of *SDT1* in our study (Table 2), which was reported to be responsible for production of precursors in NAD^+^ synthesis.^40^ Combined with the up-regulation of those genes involved in NAD(P)H generation, such as *ADH3*/*4*/*6*, *ALD6*, *TDH2*, *IDH1*/*2*, *GDH1*, *KGD1*/*2 etc.* (Fig. 2, Table 2), indicating that more NAD(P)H production could be provided. This distinctive character of enhancing NAD level in response to the multiple inhibitors may endue *K. marxianus* intrinsic considerate inhibitors tolerance especially to furfural and HMF, which was reported in our and other previous study.^13,14^ These results give us a hint that improving the amount of NAD^+^ and NADH may enhance the yeast tolerance to lignocellulosic inhibitors.

In addition, *YCT1* encoding high affinity cysteine transporter and KMAR_40340 encoding cystine transporter were up-regulated with the multiple inhibitors stress. YCT1 was reported to be the principal cysteine transporter in *S. cerevisiae*.^81^ It is well known that cysteine with reductive SH is required for the synthesis of glutathione, an essential antioxidant molecule involved in oxidative stress response and detoxification.^82^ Combined with the enhanced expression of enzyme genes at NADH/NAD^+^ shuttle sites in our study and the increased amount of NAD^+^ and NADH pool (Table 3), it once again suggest that the regeneration or conserve actual cofactors was important to remain the cellular redox balance to *K. marxianus* under the lignocellulosic inhibitors stress.

We also noticed that DEGs related to alanine, aspartate and glutamate metabolism were significantly regulated in response to the multiple inhibitors stress (Table 2). This may be explained by a previous report that the alanine, aspartate and glutamate metabolism was important for yeast cells to resist furfural, acetic acid and phenol (FAP) stress.^83^

Though SNZ1 and SNO1 were required for conditions in which vitamin B6 (pyridoxal) is essential for growth, SNZ2/SNO2 and SNZ3/SNO3 pairs seemed more related with vitamin B1 (thiamine) biosynthesis during the exponential phase in *S. cerevisiae*.^84^ In our RNA-seq results, however, there were only 2 genes encoded putative proteins showing close amino acid sequence similarity to SNZ3 and SNO3 of *S. cerevisiae*, and the transcript abundance of *SNZ3* was very high, while both *SNZ3* and *SNO3* was dramatically down-regulated in response to multiple inhibitors stress (Table 2), suggesting the encoded protein pairs may play an important role to the inhibitor tolerance in *K. marxianus*, though their precise functions in inhibitors tolerance remain to be elucidated. Furthermore, thiamine can affect metabolic functions through thiamine pyrophosphate (TPP)-dependent enzymes, such as pyruvate decarboxylase and alpha-ketoglutarate dehydrogenase which are important in the carbon central metabolism pathway and TCA cycle, respectively. In agreement with this, *PDC* coding for pyruvate decarboxylase and *KGDs* coding for alpha-ketoglutarate dehydrogenase were down or up-regulated with multiple inhibitors stress (Table 2). Meanwhile, it was reported that addition of thiamine decreased production of reactive oxygen species in yeast cells and decreases transcription of stress response genes as well.^85^ Taken together with the only pair of SNZ3 and SNO3 and high transcript abundance in our study, different from those in *S. cerevisiae*, SNZ3/SNO3 may have multiple functions in *K. marxianus*.

MSN2 and its close homolog MSN4 (referred to as MSN2/4) were identified as transcriptional activator required for expression of a wide variety of genes in response to multiple types of stress *via* interaction with the consensus sequence known as the stress responsive element (STRE) in their promoter regions.^41^ Overexpression of *MSN2* of *S. cerevisiae* confers furfural resistance in *S. cerevisiae* and expression of *MSN2* of *K. marxianus* promoted cell growth and ethanol production in *S. cerevisiae*.^86,87^ We speculate that the function of MSN2 in *K. marxianus* might not relate to the inhibitors tolerance or phosphorylation of MSN2 was more important in regulating the genes in response to the multiple inhibitors stress.

Our study reveal that the fatty acid metabolic process and ergosterol biosynthetic process were depressed by multiple fermentation inhibitors, based on the 10 DEGs involved in these two biological processes (Table 2). Previous study also showed that overexpression of *OLE1* improved the acetic acid tolerance in *S. cerevisiae*.^88^ These results pointed a hint that regulating the expression of those DEGs involved in these two processes may increase the tolerance of *K. marxianus* to the lignocellulosic inhibitors.

Interestingly, a recent report showed that iron and copper are transition metals involved in redox reactions that are essential for all eukaryotes, but whose intracellular concentrations must be carefully monitored, as they are potentially toxic.^89^ Our results showed that genes involved in iron homeostasis such as *ARN2*, *SIT1* and *FTR1*were induced while those involved in copper uptake such as *CTR1* and *FET4* were repressed under multiple inhibitors conditions. In *S. cerevisiae*, most of these genes were regulated by AFT1, a transcription factor that responds to intracellular iron.^90,91^ In our study, however, there was no change of AFT1 expression detected in transcriptional level (data not shown). Another gene KMAR_40422, encoding a probable mitochondrial transport protein FSF1 was repressed either. Interestingly, FSF1 was reported to belong to an ancient mitochondrial protein and necessary to maintain the homeostasis of iron within mitochondria.^58^ Meanwhile, from our results, up-regulation of glutamate synthesis related genes *UGA1* and *GDH1* was consistent to previous report that regulation of glutamate synthesis was dependent on the iron availability.^92^ Furthermore, the integrative analysis of the transcriptome with metabolome data revealed that the glucose metabolism, amino acid synthesis, ergosterol, and lipid biosynthesis biological processes were all affected due to the loss in the activities of specific iron-dependent enzymes under iron deprivation,^92^ and the change of expression in transcriptional level were also identified in our study (Table 2). There are several mechanisms reported on iron uptake and the regulation on overall iron homeostasis is complicated. The results in present study give us a hint that there is relationship between iron transportation and the inhibitors tolerance though the mechanism remains unclear.

Besides the multiple inhibitors stress condition to mimic the lignocellulosic biomass fermentation to study the global transcriptional response of *K. marxianus*, we also investigated some transporters transcriptional response to the three individual inhibitors stress by RT-PCR. As predicted, these genes responded quite differently to different inhibitor. For example, *ITR2* and *JEN1*, encoding myo-inositol transporter 2 and carboxylic acid transporter protein respectively, were dramatically up-regulated especially with furfural stress, even more than that with the multiple stress condition (Fig. 4). Like *MSN2* and *SNZ3/SNO3*, *ITR2* is another example of the only one isoform in *K. marxianus* in comparison to the 2 counterparts in *S. cerevisiae*. A previous study showed the essential role of *ITR2* for *Shizosaccharomyces pombe* growth.^93^ However, the regulation role of ITR2 in response to various stresses was not clear. We speculate that overexpression of *ITR2* might enhance yeast to the lignocellulosic derived inhibitors tolerance. For PDR12, with acetic acid and mixed inhibitors, it seemed to have similar up-regulation on the transcriptional expression level. In the case of *ARN2*, however, the most up-regulated stress condition was with mixed inhibitors treatment (Fig. 4). It should be noted that it is intrinsically complex and challenging to engineering yeast resistance to mixed fermentation inhibitors because each type of inhibitor may have distinct toxic effects and cellular stress response mechanisms.^94,95^

Numerous metabolic pathways and regulatory genes have been reported affecting yeast tolerance to environmental stress.^96^ It should be noted that some differentially expressed genes from RNA-seq dataset could be just passively up- or down-regulated and may not contribute to eliciting stress responses. The future work will systemically evaluate the highly ranked differentially expressed genes and identify their effects on yeast stress responses to individual inhibitor in addition to mixed fermentation inhibitors. It is known that engineering microbial resistance to fermentation inhibitors becomes even more challenging and complex as the types of inhibitors expanded in the mixture. With the transcriptomic-guided metabolic engineering approach, our future work will concentrate on characterizing the highly ranked targets functions to elicit improved resistance to multiple inhibitors.

## Conclusion

This study provides the first transcriptomic analysis of *K. marxianus* at elevated temperature (42 °C) to the multiple fermentation inhibitors stress. The results revealed that lignocellulosic hydrolysates inhibitors had effects on multiple aspects of cellular metabolism at the transcriptional level. Differentially expressed genes were enriched in maintaining the redox balance, NAD(P)^+^/NAD(P)H homeostasis or NAD^+^ synthesis, energy production, and iron transportation or metabolism. Our results suggest that redox balance and NAD(P)^+^/NAD(P)H homeostasis play an important role in tolerance to lignocellulosic derived inhibitors. Besides engineering of the redox system to relieve the stress caused by HMF and furfural, improving the level of NADH and NAD^+^ pool may represent another putative target to reduce the stress caused by the lignocellulosic derived inhibitors. Based on quite a few iron transporters were up-regulated in response to the multiple inhibitors, it hints that there is relationship between iron transportation or metabolism and the tolerance to the inhibitors though the mechanism remains unclear. The energy-consuming detoxification of furfural and HMF drove yeast to up-regulate those genes related to TCA cycle and respiratory chain to obtain more ATP. Some highly ranked differentially expressed genes were predicted as potential regulatory genes, which need further investigation. The results obtained in this study provide insights about the mechanisms of *K. marxianus* that are involved with lignocellulosic derived inhibitors response, enabling future metabolic engineering approach to obtain more robust strains for industrial fermentation with cellulosic biomass.

## Conflicts of interest

The authors declare that they have no conflict interests.

## Supplementary Material

RA-008-C8RA00335A-s001
