# Supplementary material for: Transcriptomic analysis of thermotolerant yeast Kluyveromyces marxianus in multiple inhibitors tolerance
Source: RSC Adv. 2018 Apr 17;8(26):14177–92. doi: 10.1039/c8ra00335a (PMC9079866; doi:10.1039/c8ra00335a)
Supplement: RA-008-C8RA00335A-s001 [file RA-008-C8RA00335A-s001.pdf]

Electronic Supplementary Material (ESI) for RSC Advances.
This journal is © The Royal Society of Chemistry 2018

Electronic Supplementary Information

Transcriptomic analysis of thermotolerant yeast *Kluyveromyces marxianus* in multiple inhibitors tolerance

Dongmei Wang, Dan Wu, Xiaoxue Yang, Jiong Hong *

Affiliation:

School of Life Sciences, University of Science and Technology of China, Hefei, Anhui, 230027, P. R. China

* Corresponding author.

Tel.: +86 551 63600705; Fax: +86 551 63601443;

E-mail address: hjiong@ustc.edu.cn

Table S1 Summary of mapping results from RNA-seq of *K. marxianus*

Sample	Clean reads	Clean base (bp)	> Q20 (%)	> Q30 (%)	GC (%)	Uniquely Mapping reads	Mapping rate (%)
I	13,622,794	2.44E+09	97.68	92.79	44.57	12,490,287	91.69
C	17,697,296	1.89E+09	97.31	91.87	46.27	16,279,803	91.99

Note: I and C represent samples of yeast grown on medium with or without multiple fermentation inhibitors in YPD under aerobic condition. Q20, Q30 refer to the proportion of base calls with Phred scores >20 or 30 in the total bases.

Table S2 Summary of differentially expressed genes involved in GO enrichments in multiple inhibitors treated condition.

ID	Description	DEGs in study	Background	Ratio in background	Number up	Number down
GO:0006096	glycolytic process	16	26	0.62	2	14
GO:0032787	monocarboxylic acid metabolic process	40	90	0.44	13	27
GO:0006090	pyruvate metabolic process	22	42	0.52	6	16
GO:0006094	gluconeogenesis	17	31	0.55	3	14
GO:0006091	generation of precursor metabolites and energy	27	71	0.38	11	16
GO:0055114	oxidation-reduction process	69	304	0.23	33	36
GO:0016491	oxidoreductase activity	65	286	0.23	36	29
GO:0043436	oxoacid metabolic process	77	394	0.20	26	51
GO:0006082	organic acid metabolic process	77	395	0.19	26	51
GO:0019319	hexose biosynthetic process	17	32	0.53	3	14
GO:0046364	monosaccharide biosynthetic process	17	32	0.53	3	14
GO:0044281	small molecule metabolic process	115	689	0.17	42	73
GO:0019752	carboxylic acid metabolic process	77	393	0.20	26	51
GO:0044763	single-organism cellular process	184	1329	0.14	89	95
GO:0044710	single-organism metabolic process	171	1138	0.15	80	91
GO:0006099	tricarboxylic acid cycle	16	29	0.55	16	0
GO:0044699	single-organism process	220	1602	0.14	113	107
GO:0044711	single-organism biosynthetic process	64	344	0.19	12	52
GO:0006006	glucose metabolic process	18	43	0.42	3	15
GO:0003824	catalytic activity	212	1754	0.12	112	100
GO:1901564	organonitrogen compound metabolic process	98	641	0.15	34	64
GO:0016051	carbohydrate biosynthetic process	18	47	0.38	3	15
GO:0044724	single-organism carbohydrate catabolic process	17	43	0.40	2	15
GO:0016614	oxidoreductase activity, acting on CH-OH group of donors	18	51	0.35	9	9

GO:0016052	carbohydrate catabolic process	17	47	0.36	2	15
GO:0019318	hexose metabolic process	19	58	0.33	4	15
GO:0016616	oxidoreductase activity, acting on the CH-OH group of donors, NAD or NADP as acceptor	17	48	0.35	8	9
GO:0005996	monosaccharide metabolic process	19	60	0.32	4	15
GO:0008152	metabolic process	243	2176	0.11	126	117
GO:0044262	cellular carbohydrate metabolic process	17	50	0.34	7	10
GO:0055085	transmembrane transport	49	269	0.18	35	14
GO:0048037	cofactor binding	31	138	0.22	16	15
GO:0009987	cellular process	230	2047	0.11	118	112
GO:1990204	oxidoreductase complex	10	20	0.50	10	0
GO:0051186	cofactor metabolic process	27	118	0.23	7	20
GO:0015976	carbon utilization	8	13	0.62	4	4
GO:0046487	glyoxylate metabolic process	8	13	0.62	6	2
GO:0005975	carbohydrate metabolic process	35	178	0.20	14	21
GO:1901576	organic substance biosynthetic process	84	599	0.14	18	66
GO:0044712	single-organism catabolic process	39	214	0.18	11	28
GO:0009058	biosynthetic process	86	626	0.14	19	67
GO:0006732	coenzyme metabolic process	22	93	0.24	5	17
GO:0044723	single-organism carbohydrate metabolic process	27	128	0.21	9	18
GO:0003674	molecular_function	291	2817	0.10	166	125
GO:0044237	cellular metabolic process	181	1595	0.11	82	99
GO:1901566	organonitrogen compound biosynthetic process	39	223	0.17	8	31
GO:0051287	NAD binding	9	21	0.43	4	5
GO:0006081	cellular aldehyde metabolic process	9	21	0.43	6	3
GO:1901070	guanosine-containing compound biosynthetic process	4	4	1.00	1	3
GO:0008150	biological_process	314	3115	0.10	180	134
GO:0044283	small molecule biosynthetic process	33	185	0.18	5	28

GO:0050662	coenzyme binding	20	91	0.22	10	10
GO:0071704	organic substance metabolic process	186	1677	0.11	81	105
GO:0006740	NADPH regeneration	8	19	0.42	1	7
GO:0006007	glucose catabolic process	8	19	0.42	1	7
GO:0019320	hexose catabolic process	8	19	0.42	1	7
GO:0006098	pentose-phosphate shunt	8	19	0.42	1	7
GO:0006753	nucleoside phosphate metabolic process	37	222	0.17	14	23
GO:0005985	sucrose metabolic process	7	15	0.47	3	4
GO:0005982	starch metabolic process	7	15	0.47	3	4
GO:0043169	cation binding	73	543	0.13	41	32
GO:0043167	ion binding	132	1127	0.12	73	59
GO:0009117	nucleotide metabolic process	36	218	0.17	13	23
GO:0046872	metal ion binding	72	539	0.13	40	32
GO:0006000	fructose metabolic process	7	16	0.44	2	5
GO:0006013	mannose metabolic process	7	16	0.44	2	5
GO:0006739	NADP metabolic process	8	21	0.38	1	7
GO:0046365	monosaccharide catabolic process	8	21	0.38	1	7
GO:0009152	purine ribonucleotide biosynthetic process	10	32	0.31	3	7
GO:0006164	purine nucleotide biosynthetic process	10	32	0.31	3	7
GO:0046496	nicotinamide nucleotide metabolic process	9	27	0.33	2	7
GO:0005984	disaccharide metabolic process	7	17	0.41	3	4
GO:0009063	cellular amino acid catabolic process	8	22	0.36	3	5
GO:0009260	ribonucleotide biosynthetic process	11	40	0.28	3	8
GO:0046912	transferase activity, transferring acyl groups, acyl groups converted into alkyl on transfer	5	9	0.56	2	3
GO:0006790	sulfur compound metabolic process	15	67	0.22	3	12
GO:0009168	purine ribonucleoside monophosphate biosynthetic process	9	29	0.31	2	7
GO:0009127	purine nucleoside monophosphate biosynthetic process	9	29	0.31	2	7

GO:0000287	magnesium ion binding	11	41	0.27	3	8
GO:0046390	ribose phosphate biosynthetic process	11	41	0.27	3	8
GO:0046037	GMP metabolic process	3	3	1.00	0	3
GO:0004365	glyceraldehyde-3-phosphate dehydrogenase (NAD+) (phosphorylating) activity	3	3	1.00	1	2
GO:0006177	GMP biosynthetic process	3	3	1.00	0	3
GO:0046395	carboxylic acid catabolic process	8	24	0.33	3	5
GO:0016054	organic acid catabolic process	8	24	0.33	3	5
GO:0072522	purine-containing compound biosynthetic process	10	36	0.28	3	7
GO:0006144	purine nucleobase metabolic process	16	77	0.21	4	12
GO:0006733	oxidoreduction coenzyme metabolic process	10	37	0.27	2	8
GO:0019362	pyridine nucleotide metabolic process	9	31	0.29	2	7
GO:0055086	nucleobase-containing small molecule metabolic process	41	281	0.15	16	25
GO:0006073	cellular glucan metabolic process	7	20	0.35	3	4
GO:1901606	alpha-amino acid catabolic process	7	20	0.35	3	4
GO:0009311	oligosaccharide metabolic process	7	20	0.35	3	4
GO:0044042	glucan metabolic process	7	20	0.35	3	4
GO:0072524	pyridine-containing compound metabolic process	10	38	0.26	2	8
GO:0005829	cytosol	8	26	0.31	4	4
GO:0009167	purine ribonucleoside monophosphate metabolic process	21	117	0.18	10	11
GO:0009126	purine nucleoside monophosphate metabolic process	21	117	0.18	10	11
GO:0008483	transaminase activity	7	21	0.33	3	4
GO:0044264	cellular polysaccharide metabolic process	7	21	0.33	3	4
GO:0005976	polysaccharide metabolic process	7	21	0.33	3	4

GO:0016769	transferase activity, transferring nitrogenous groups	7	21	0.33	3	4
GO:0044765	single-organism transport	53	394	0.13	37	16
GO:0045275	respiratory chain complex III	4	7	0.57	3	0
GO:0006553	lysine metabolic process	6	16	0.38	2	4
GO:0072330	monocarboxylic acid biosynthetic process	8	27	0.30	1	7
GO:0051188	cofactor biosynthetic process	14	67	0.21	5	9
GO:0046394	carboxylic acid biosynthetic process	25	151	0.17	4	21
GO:0016053	organic acid biosynthetic process	25	151	0.17	4	21
GO:0009156	ribonucleoside monophosphate biosynthetic process	10	40	0.25	2	8
GO:0044238	primary metabolic process	171	1604	0.11	79	92
GO:0009161	ribonucleoside monophosphate metabolic process	22	128	0.17	10	12
GO:0006520	cellular amino acid metabolic process	45	325	0.14	14	31
GO:0009123	nucleoside monophosphate metabolic process	22	130	0.17	10	12
GO:0004022	alcohol dehydrogenase (NAD) activity	3	4	0.75	2	1
GO:1901659	glycosyl compound biosynthetic process	9	35	0.26	3	6
GO:0009081	branched-chain amino acid metabolic process	7	23	0.30	0	7
GO:0044282	small molecule catabolic process	8	29	0.28	3	5
GO:0070013	intracellular organelle lumen	8	29	0.28	8	0
GO:0043233	organelle lumen	8	29	0.28	8	0
GO:0019637	organophosphate metabolic process	41	294	0.14	15	26
GO:0009124	nucleoside monophosphate biosynthetic process	10	42	0.24	2	8
GO:0009066	aspartate family amino acid metabolic process	15	77	0.19	3	12
GO:0006119	oxidative phosphorylation	6	18	0.33	5	1
GO:0016209	antioxidant activity	6	18	0.33	4	2

GO:0051539	4 iron, 4 sulfur cluster binding	5	13	0.38	3	2
GO:0016021	integral component of membrane	60	479	0.13	41	19
GO:0044425	membrane part	74	617	0.12	51	23
GO:0006793	phosphorus metabolic process	61	487	0.13	24	37
GO:0009112	nucleobase metabolic process	17	93	0.18	4	13
GO:0046129	purine ribonucleoside biosynthetic process	7	25	0.28	3	4
GO:0042451	purine nucleoside biosynthetic process	7	25	0.28	3	4
GO:0031974	membrane-enclosed lumen	9	38	0.24	9	0
GO:1901362	organic cyclic compound biosynthetic process	34	240	0.14	9	25
GO:0034614	cellular response to reactive oxygen species	4	9	0.44	2	2
GO:0022857	transmembrane transporter activity	28	188	0.15	19	9
GO:0020037	heme binding	8	32	0.25	1	7
GO:0046906	tetrapyrrole binding	8	32	0.25	1	7
GO:0009163	nucleoside biosynthetic process	8	32	0.25	3	5
GO:0042455	ribonucleoside biosynthetic process	8	32	0.25	3	5
GO:0009056	catabolic process	42	315	0.13	13	29
GO:0043604	amide biosynthetic process	7	26	0.27	1	6
GO:0005215	transporter activity	33	232	0.14	23	10
GO:0042723	thiamine-containing compound metabolic process	3	5	0.60	0	3
GO:1990351	transporter complex	3	5	0.60	3	0
GO:0030976	thiamine pyrophosphate binding	3	5	0.60	2	1
GO:0006772	thiamine metabolic process	3	5	0.60	0	3
GO:0070469	respiratory chain	3	5	0.60	3	0
GO:1902495	transmembrane transporter complex	3	5	0.60	3	0
GO:0044249	cellular biosynthetic process	69	573	0.12	15	54
GO:0016836	hydro-lyase activity	6	20	0.30	4	2
GO:0006796	phosphate-containing compound metabolic process	59	479	0.12	23	36

GO:1901605	alpha-amino acid metabolic process	39	289	0.13	11	28
GO:0006066	alcohol metabolic process	7	27	0.26	0	7
GO:0009108	coenzyme biosynthetic process	11	55	0.20	3	8
GO:0004449	isocitrate dehydrogenase (NAD+) activity	2	2	1.00	2	0
GO:0003872	6-phosphofructokinase activity	2	2	1.00	0	2
GO:0006002	fructose 6-phosphate metabolic process	2	2	1.00	0	2
GO:0016801	hydrolase activity, acting on ether bonds	2	2	1.00	0	2
GO:0016805	dipeptidase activity	2	2	1.00	0	2
GO:0070283	radical SAM enzyme activity	2	2	1.00	1	1
GO:1903289	regulation of ATP catabolic process	2	2	1.00	2	0
GO:0004410	homocitrate synthase activity	2	2	1.00	0	2
GO:0043171	peptide catabolic process	2	2	1.00	0	2
GO:0006510	ATP-dependent proteolysis	2	2	1.00	2	0
GO:0004176	ATP-dependent peptidase activity	2	2	1.00	2	0
GO:0004347	glucose-6-phosphate isomerase activity	2	2	1.00	0	2
GO:0032781	positive regulation of ATPase activity	2	2	1.00	2	0
GO:0003938	IMP dehydrogenase activity	2	2	1.00	0	2
GO:0001671	ATPase activator activity	2	2	1.00	2	0
GO:0043462	regulation of ATPase activity	2	2	1.00	2	0
GO:0005945	6-phosphofructokinase complex	2	2	1.00	0	2
GO:0006633	fatty acid biosynthetic process	6	21	0.29	1	5
GO:0016765	transferase activity, transferring alkyl or aryl (other than methyl) groups	6	21	0.29	1	5
GO:0044445	cytosolic part	6	21	0.29	0	6
GO:1901615	organic hydroxy compound metabolic process	8	34	0.24	0	8
GO:0009110	vitamin biosynthetic process	9	41	0.22	1	8
GO:0042364	water-soluble vitamin	9	41	0.22	1	8

	biosynthetic process					
GO:0030170	pyridoxal phosphate binding	9	41	0.22	4	5
GO:0072593	reactive oxygen species metabolic process	4	10	0.40	2	2
GO:0008299	isoprenoid biosynthetic process	4	10	0.40	0	4
GO:0000302	response to reactive oxygen species	4	10	0.40	2	2
GO:1901701	cellular response to oxygen-containing compound	4	10	0.40	2	2
GO:0006720	isoprenoid metabolic process	4	10	0.40	0	4
GO:0016684	oxidoreductase activity, acting on peroxide as acceptor	4	10	0.40	2	2
GO:0019751	polyol metabolic process	4	10	0.40	0	4
GO:0044271	cellular nitrogen compound biosynthetic process	32	230	0.14	9	23
GO:0006631	fatty acid metabolic process	7	28	0.25	1	6
GO:0051536	iron-sulfur cluster binding	7	28	0.25	5	2
GO:0051540	metal cluster binding	7	28	0.25	5	2
GO:0006766	vitamin metabolic process	12	61	0.20	2	10
GO:0006767	water-soluble vitamin metabolic process	12	61	0.20	2	10
GO:0022891	substrate-specific transmembrane transporter activity	24	159	0.15	15	9
GO:0042221	response to chemical	8	35	0.23	5	3
GO:1901575	organic substance catabolic process	38	287	0.13	11	27
GO:0050661	NADP binding	6	22	0.27	1	5
GO:0016903	oxidoreductase activity, acting on the aldehyde or oxo group of donors	6	22	0.27	3	3
GO:0006163	purine nucleotide metabolic process	24	163	0.15	11	13
GO:0072521	purine-containing compound metabolic process	31	225	0.14	14	17
GO:0019878	lysine biosynthetic process via aminoadipic acid	3	6	0.50	0	3
GO:0045239	tricarboxylic acid cycle enzyme complex	3	6	0.50	3	0
GO:0051087	chaperone binding	3	6	0.50	3	0
GO:1901681	sulfur compound binding	3	6	0.50	2	1

GO:1901700	response to oxygen-containing compound	4	11	0.36	2	2
GO:0009085	lysine biosynthetic process	4	11	0.36	0	4
GO:0010035	response to inorganic substance	4	11	0.36	2	2
GO:0006694	steroid biosynthetic process	5	17	0.29	0	5
GO:0016620	oxidoreductase activity, acting on the aldehyde or oxo group of donors, NAD or NADP as acceptor	5	17	0.29	2	3
GO:0006807	nitrogen compound metabolic process	115	1071	0.11	47	68
GO:0016835	carbon-oxygen lyase activity	6	24	0.25	4	2
GO:0005759	mitochondrial matrix	6	24	0.25	6	0
GO:0009165	nucleotide biosynthetic process	11	58	0.19	3	8
GO:0034599	cellular response to oxidative stress	5	18	0.28	3	2
GO:0008202	steroid metabolic process	5	18	0.28	0	5
GO:1901293	nucleoside phosphate biosynthetic process	11	60	0.18	3	8
GO:0031072	heat shock protein binding	3	7	0.43	3	0
GO:0044272	sulfur compound biosynthetic process	8	39	0.21	2	6
GO:0009259	ribonucleotide metabolic process	24	170	0.14	11	13
GO:0008643	carbohydrate transport	6	25	0.24	4	2
GO:0005739	mitochondrion	16	101	0.16	11	5
GO:0019693	ribose phosphate metabolic process	24	172	0.14	11	13
GO:0016829	lyase activity	11	62	0.18	7	4
GO:0072528	pyrimidine-containing compound biosynthetic process	5	19	0.26	1	4
GO:0006551	leucine metabolic process	5	19	0.26	0	5
GO:0016310	phosphorylation	27	200	0.14	13	14
GO:0009266	response to temperature stimulus	2	3	0.67	2	0
GO:0016635	oxidoreductase activity, acting on the CH-CH group of donors, quinone or related compound as acceptor	2	3	0.67	1	1
GO:0060590	ATPase regulator activity	2	3	0.67	2	0
GO:0004888	transmembrane signaling	2	3	0.67	2	0

	receptor activity					
GO:0003984	acetolactate synthase activity	2	3	0.67	0	2
GO:0045252	oxoglutarate dehydrogenase complex	2	3	0.67	2	0
GO:0004096	catalase activity	2	3	0.67	0	2
GO:0003994	aconitate hydratase activity	2	3	0.67	2	0
GO:0045281	succinate dehydrogenase complex	2	3	0.67	2	0
GO:0045240	dihydrolipoyl dehydrogenase complex	2	3	0.67	2	0
GO:0042744	hydrogen peroxide catabolic process	2	3	0.67	0	2
GO:0009408	response to heat	2	3	0.67	2	0
GO:0009083	branched-chain amino acid catabolic process	2	3	0.67	0	2
GO:0000104	succinate dehydrogenase activity	2	3	0.67	2	0
GO:0008612	peptidyl-lysine modification to peptidyl-hypusine	2	3	0.67	0	2
GO:0005750	mitochondrial respiratory chain complex III	2	3	0.67	2	0
GO:0004488	methylenetetrahydrofolate dehydrogenase (NADP+) activity	2	3	0.67	0	2
GO:0006097	glyoxylate cycle	2	3	0.67	2	0
GO:0006552	leucine catabolic process	2	3	0.67	0	2
GO:0005948	acetolactate synthase complex	2	3	0.67	0	2
GO:0016860	intramolecular oxidoreductase activity	4	13	0.31	1	3
GO:0042398	cellular modified amino acid biosynthetic process	7	33	0.21	1	6
GO:0070887	cellular response to chemical stimulus	6	26	0.23	4	2
GO:0009150	purine ribonucleotide metabolic process	23	162	0.14	11	12
GO:0005506	iron ion binding	7	34	0.21	2	5
GO:0009082	branched-chain amino acid biosynthetic process	5	20	0.25	0	5
GO:0008204	ergosterol metabolic process	3	8	0.38	0	3
GO:0004659	prenyltransferase activity	3	8	0.38	0	3
GO:0051537	2 iron, 2 sulfur cluster binding	3	8	0.38	2	1

GO:0016776	phosphotransferase activity, phosphate group as acceptor	3	8	0.38	1	2
GO:0016128	phytosteroid metabolic process	3	8	0.38	0	3
GO:0016129	phytosteroid biosynthetic process	3	8	0.38	0	3
GO:0019842	vitamin binding	3	8	0.38	2	1
GO:0006696	ergosterol biosynthetic process	3	8	0.38	0	3
GO:0006189	'de novo' IMP biosynthetic process	3	8	0.38	0	3
GO:0006541	glutamine metabolic process	3	8	0.38	0	3
GO:0044107	cellular alcohol metabolic process	3	8	0.38	0	3
GO:0044108	cellular alcohol biosynthetic process	3	8	0.38	0	3
GO:0019643	reductive tricarboxylic acid cycle	4	14	0.29	4	0
GO:0015977	carbon fixation	4	14	0.29	4	0
GO:0018130	heterocycle biosynthetic process	29	223	0.13	9	20
GO:0022892	substrate-specific transporter activity	24	177	0.14	15	9
GO:0008238	exopeptidase activity	6	28	0.21	2	4
GO:0051082	unfolded protein binding	6	28	0.21	6	0
GO:1901617	organic hydroxy compound biosynthetic process	5	21	0.24	0	5
GO:0006549	isoleucine metabolic process	5	21	0.24	0	5
GO:1901137	carbohydrate derivative biosynthetic process	12	74	0.16	3	9
GO:0006457	protein folding	9	49	0.18	8	1
GO:0046165	alcohol biosynthetic process	4	15	0.27	0	4
GO:0004497	monooxygenase activity	4	15	0.27	0	4
GO:0008652	cellular amino acid biosynthetic process	18	126	0.14	3	15
GO:0046040	IMP metabolic process	3	9	0.33	0	3
GO:0006730	one-carbon metabolic process	3	9	0.33	1	2
GO:0006188	IMP biosynthetic process	3	9	0.33	0	3
GO:0016861	intramolecular oxidoreductase activity, interconverting aldoses and ketoses	3	9	0.33	0	3
GO:0006575	cellular modified amino acid	9	50	0.18	1	8

	metabolic process					
GO:0030554	adenyl nucleotide binding	53	462	0.11	30	23
GO:0016830	carbon-carbon lyase activity	5	22	0.23	3	2
GO:0036094	small molecule binding	78	718	0.11	39	39
GO:0006020	inositol metabolic process	2	4	0.50	0	2
GO:0042724	thiamine-containing compound biosynthetic process	2	4	0.50	0	2
GO:0004448	isocitrate dehydrogenase activity	2	4	0.50	2	0
GO:0043446	cellular alkane metabolic process	2	4	0.50	0	2
GO:0006108	malate metabolic process	2	4	0.50	2	0
GO:0016615	malate dehydrogenase activity	2	4	0.50	2	0
GO:0009228	thiamine biosynthetic process	2	4	0.50	0	2
GO:0015947	methane metabolic process	2	4	0.50	0	2
GO:0004784	superoxide dismutase activity	2	4	0.50	2	0
GO:0042743	hydrogen peroxide metabolic process	2	4	0.50	0	2
GO:0016721	oxidoreductase activity, acting on superoxide radicals as acceptor	2	4	0.50	2	0
GO:0008144	drug binding	2	4	0.50	0	2
GO:0018065	protein-cofactor linkage	2	4	0.50	2	0
GO:0016114	terpenoid biosynthetic process	2	4	0.50	0	2
GO:0006515	misfolded or incompletely synthesized protein catabolic process	2	4	0.50	2	0
GO:0006721	terpenoid metabolic process	2	4	0.50	0	2
GO:0008121	ubiquinol-cytochrome-c reductase activity	2	4	0.50	2	0
GO:0016681	oxidoreductase activity, acting on diphenols and related substances as donors, cytochrome as acceptor	2	4	0.50	2	0
GO:0016679	oxidoreductase activity, acting on diphenols and related substances as donors	2	4	0.50	2	0
GO:0009071	serine family amino acid catabolic process	2	4	0.50	0	2

GO:0070301	cellular response to hydrogen peroxide	2	4	0.50	0	2
GO:0006546	glycine catabolic process	2	4	0.50	0	2
GO:0006563	L-serine metabolic process	6	30	0.20	1	5
GO:0043603	cellular amide metabolic process	9	52	0.17	1	8
GO:0005737	cytoplasm	33	266	0.12	8	25
GO:0015940	pantothenate biosynthetic process	4	16	0.25	1	3
GO:0015939	pantothenate metabolic process	4	16	0.25	1	3
GO:0016740	transferase activity	67	607	0.11	30	37
GO:0006950	response to stress	22	164	0.13	19	3

Note: Ratio in background represents the ratio of DEGs in the specific GO categaries (background genes). Number up or down represent the number of DEG up- or down-regulated in response to the multiple inhibitors stress.

Table S3 Summary of differentially expressed genes involved in KEGG pathways in multiple inhibitors treated condition.

ID	Term	DEGs	Background genes	DEGs/backgroud genes
ko01220	Degradation of aromatic compounds	4	6	0.667
ko00830	Retinol metabolism	3	5	0.600
ko00983	Drug metabolism - other enzymes	4	7	0.571
ko00010	Glycolysis / Gluconeogenesis	25	44	0.568
ko00680	Methane metabolism	11	20	0.550
ko00710	Carbon fixation in photosynthetic organisms	9	17	0.529
ko00020	Citrate cycle (TCA cycle)	14	28	0.500
ko00630	Glyoxylate and dicarboxylate metabolism	10	20	0.500
ko00130	Ubiquinone and other terpenoid-quinone biosynthesis	5	10	0.500
ko00625	Chloroalkane and chloroalkene degradation	4	8	0.500
ko00626	Naphthalene degradation	3	6	0.500
ko00300	Lysine biosynthesis	6	13	0.462
ko01200	Carbon metabolism	42	92	0.457
ko01210	2-Oxocarboxylic acid metabolism	15	35	0.429
ko00730	Thiamine metabolism	3	7	0.429
ko04626	Plant-pathogen interaction	2	5	0.400
ko04612	Antigen processing and presentation	2	5	0.400
ko04621	NOD-like receptor signaling pathway	2	5	0.400
ko00950	Isoquinoline alkaloid biosynthesis	2	5	0.400
ko00521	Streptomycin biosynthesis	2	5	0.400
ko00030	Pentose phosphate pathway	9	23	0.391
ko00500	Starch and sucrose metabolism	12	31	0.387
ko00052	Galactose metabolism	5	13	0.385
ko00380	Tryptophan metabolism	6	16	0.375
ko00750	Vitamin B6 metabolism	3	8	0.375
ko00280	Valine, leucine and isoleucine degradation	4	11	0.364
ko00650	Butanoate metabolism	4	11	0.364
ko05230	Central carbon metabolism in cancer	5	14	0.357
ko00051	Fructose and mannose metabolism	6	17	0.353
ko00910	Nitrogen metabolism	2	6	0.333
ko00260	Glycine, serine and threonine metabolism	10	32	0.313
ko00670	One carbon pool by folate	5	16	0.313
ko00290	Valine, leucine and isoleucine biosynthesis	4	13	0.308
ko00980	Metabolism of xenobiotics by cytochrome P450	3	10	0.300
ko00982	Drug metabolism - cytochrome P450	3	10	0.300
ko01230	Biosynthesis of amino acids	33	112	0.295
ko00071	Fatty acid degradation	5	17	0.294
ko00350	Tyrosine metabolism	5	17	0.294
ko00780	Biotin metabolism	2	7	0.286

ko00620	Pyruvate metabolism	11	39	0.282
ko04152	AMPK signaling pathway	7	25	0.280
ko04066	HIF-1 signaling pathway	5	18	0.278
ko00040	Pentose and glucuronate interconversions	3	11	0.273
ko04260	Cardiac muscle contraction	5	20	0.250
ko04068	FoxO signaling pathway	4	16	0.250
ko00770	Pantothenate and CoA biosynthesis	4	16	0.250
ko00627	Aminobenzoate degradation	3	12	0.250
ko00190	Oxidative phosphorylation	14	58	0.241
ko00900	Terpenoid backbone biosynthesis	4	17	0.235
ko00740	Riboflavin metabolism	3	13	0.231
ko00360	Phenylalanine metabolism	3	13	0.231
ko00960	Tropane, piperidine and pyridine alkaloid biosynthesis	2	9	0.222
ko03320	PPAR signaling pathway	2	9	0.222
ko00250	Alanine, aspartate and glutamate metabolism	5	24	0.208
ko02020	Two-component system	3	15	0.200
ko00410	beta-Alanine metabolism	3	15	0.200
ko00100	Steroid biosynthesis	3	15	0.200
ko04727	GABAergic synapse	2	10	0.200
ko00903	Limonene and pinene degradation	1	5	0.200
ko00860	Porphyrin and chlorophyll metabolism	3	16	0.188
ko00310	Lysine degradation	3	16	0.188
ko00720	Carbon fixation pathways in prokaryotes	2	11	0.182
ko04915	Estrogen signaling pathway	2	11	0.182
ko04146	Peroxisome	7	39	0.179
ko04910	Insulin signaling pathway	4	23	0.174
ko00561	Glycerolipid metabolism	4	23	0.174
ko00270	Cysteine and methionine metabolism	6	36	0.167
ko04622	RIG-I-like receptor signaling pathway	1	6	0.167
ko00520	Amino sugar and nucleotide sugar metabolism	4	25	0.160
ko00230	Purine metabolism	13	87	0.149
ko03010	Ribosome	11	76	0.145
ko00480	Glutathione metabolism	3	21	0.143
ko00061	Fatty acid biosynthesis	1	7	0.143
ko04920	Adipocytokine signaling pathway	1	7	0.143
ko00460	Cyanoamino acid metabolism	1	7	0.143
ko00330	Arginine and proline metabolism	5	36	0.139
ko03430	Mismatch repair	3	22	0.136
ko00640	Propanoate metabolism	2	16	0.125
ko00600	Sphingolipid metabolism	2	16	0.125
ko04122	Sulfur relay system	1	8	0.125
ko04918	Thyroid hormone synthesis	1	8	0.125
ko04140	Regulation of autophagy	2	17	0.118
ko04142	Lysosome	2	17	0.118

ko04071	Sphingolipid signaling pathway	2	17	0.118
ko00790	Folate biosynthesis	1	9	0.111
ko03450	Non-homologous end-joining	1	9	0.111
ko00450	Selenocompound metabolism	1	9	0.111
ko04151	PI3K-Akt signaling pathway	2	20	0.100
ko01212	Fatty acid metabolism	2	21	0.095
ko04020	Calcium signaling pathway	1	11	0.091
ko01040	Biosynthesis of unsaturated fatty acids	1	12	0.083
ko00514	Other types of O-glycan biosynthesis	1	12	0.083
ko04022	cGMP-PKG signaling pathway	1	12	0.083
ko04966	Collecting duct acid secretion	1	12	0.083
ko00340	Histidine metabolism	1	12	0.083
ko03018	RNA degradation	4	49	0.082
ko04141	Protein processing in endoplasmic reticulum	6	76	0.079
ko00920	Sulfur metabolism	1	13	0.077
ko04666	Fc gamma R-mediated phagocytosis	1	13	0.077
ko00562	Inositol phosphate metabolism	1	13	0.077
ko04144	Endocytosis	3	40	0.075
ko04113	Meiosis - yeast	7	99	0.071
ko03040	Spliceosome	4	63	0.063
ko00760	Nicotinate and nicotinamide metabolism	1	16	0.063
ko03022	Basal transcription factors	2	32	0.063
ko00564	Glycerophospholipid metabolism	2	33	0.061
ko03440	Homologous recombination	1	18	0.056
ko03460	Fanconi anemia pathway	1	18	0.056
ko04810	Regulation of actin cytoskeleton	1	18	0.056
ko03060	Protein export	1	18	0.056
ko04010	MAPK signaling pathway	1	18	0.056
ko00400	Phenylalanine, tyrosine and tryptophan biosynthesis	1	19	0.053
ko04921	Oxytocin signaling pathway	1	19	0.053
ko04914	Progesterone-mediated oocyte maturation	1	19	0.053
ko04721	Synaptic vesicle cycle	1	22	0.045
ko00240	Pyrimidine metabolism	3	68	0.044
ko00513	Various types of N-glycan biosynthesis	1	32	0.031
ko04145	Phagosome	1	32	0.031
ko03420	Nucleotide excision repair	1	36	0.028
ko04111	Cell cycle - yeast	3	110	0.027
ko04011	MAPK signaling pathway - yeast	1	37	0.027
ko00970	Aminoacyl-tRNA biosynthesis	1	38	0.026
ko03013	RNA transport	1	79	0.013

Note: The ratio of DEGs/background genes indicates the effects of the DEGs in the specific KEGG pathway (background genes).

Table S4 Primers used in this study.

ID	Gene	NR_Description	Primer	Sequence (5'→3')
KMAR_10772		uncharacterized abhydrolase domain-containing protein YGR015C	Forward	CCCTTGTGAAATCTTGCGTTAAGTTG
			Reverse	AATGTAGCATACCGTCGTCATAGTTG
KMAR_80057	*RPB1*	DNA-directed RNA polymerase II subunit RPB1	Forward	CCGTGGTAACACTCCATTCTTCAAG
			Reverse	GAGCAGAGGTAGCGTTGTAAGTTG
KMAR_50053	*IRC8*	uncharacterized protein IRC8	Forward	GCAACAGAAACACTCACCACAAAG
			Reverse	TCCCTTCCCGCACCGAAAC
KMAR_10795	*NQO2*	ribosyldihydronicotinamide dehydrogenase	Forward	GCACCCAGAATTAAGTGATCCAGAAG
			Reverse	ACATTCAAACCCGAGTAACCGAAAG
KMAR_80139		carbonic anhydrase	Forward	TGAAGGTGAACAAAGTCGTCATCTG
			Reverse	TCGTCGAGGTCCTGGAGGTAG
KMAR_50521	*SDH4*	succinate dehydrogenase	Forward	CCACTCTCGCTTCCGTCCTATTG
			Reverse	CCCATGATACCACCTTCCTTTGTTTC
KMAR_10054	*HBN1*	putative nitroreductase HBN1	Forward	GGCTTCTGTTACCGATGCTGTTG
			Reverse	TGGATTGGTCTGCGAAGGATGG
KMAR_20313	*MCH5*	riboflavin transporter MCH5	Forward	ACACTCTTAACCTTCGTCAT
			Reverse	GGCACCAGATATAGGAACT
KMAR_70277	*CTR1*	copper transport protein CTR1	Forward	CCGTCTCTTAATCACATTCG
			Reverse	CCATCGTCATCTTCATAGGA
KMAR_40093	*ARN2*	siderophore iron transporter ARN2	Forward	TTCAGAAGCGGTAAGGATT
			Reverse	CGAGGTGATGGATTGGAT
KMAR_10790	*SIT1*	major facilitator superfamily	Forward	CTATCTGGACCTCTGAACTT
			Reverse	AATAGAACTGACACCAACAC
KMAR_30337	*PDR12*	ATP-dependent permease PDR12	Forward	CTGCCTACTTCGTGTCTTA
			Reverse	CACAGAACAACAACATACCA
KMAR_80266	*ITR2*	myo-inositol transporter 2	Forward	CTTCTGCTACTTGTGTTATCC
			Reverse	TCTCTTCTTGGCTAGTTGTT
KMAR_20248	*JEN2*	putative sialic acid transporter	Forward	GTGCTTCCTCTACGATTGA
			Reverse	CAGGTCCTAGAATGGTAATGA
KMAR_60075	*JEN1*	carboxylic acid transporter protein homolog	Forward	AAGAAGGATTACGCTAAGGT
			Reverse	GAGATGGATAGACTGGAGAC
KMAR_50130	*FNX1*	multidrug resistance protein fnx1	Forward	CGGTCTAATGGCAATAGGA
			Reverse	AGCGAGAAGAGTAACAGTAA
KMAR_70043	*ACT1*	Actin	Forward	TTGGCTGGTAGAGACATCACTGAC
			Reverse	AGCAGATGATTGAGAAGCGGTTTG

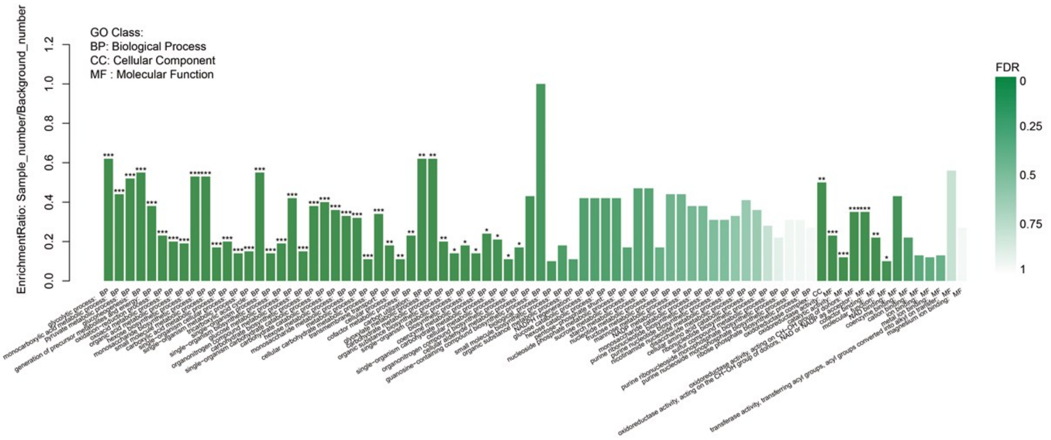

Figure S1 Differentially expressed genes in GO enrichments in multiple inhibitors treated condition.

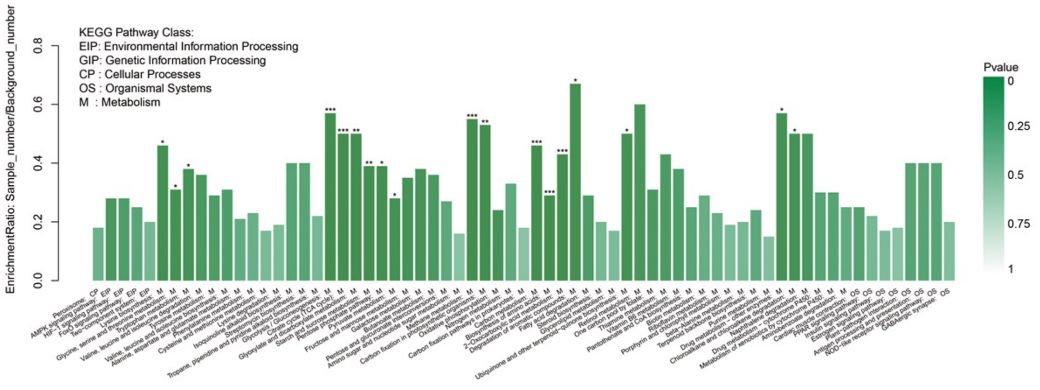

Figure S2 Differentially expressed genes in KEGG pathways in multiple inhibitors treated condition.
